# CPGAVAS2, an integrated plastome sequence annotator and analyzer

**DOI:** 10.1093/nar/gkz345

**Published:** 2019-05-08

**Authors:** Linchun Shi, Haimei Chen, Mei Jiang, Liqiang Wang, Xi Wu, Linfang Huang, Chang Liu

**Affiliations:** Key Laboratory of Bioactive Substances and Resource Utilization of Chinese Herbal Medicine from Ministry of Education, Institute of Medicinal Plant Development, Chinese Academy of Medical Sciences and Peking Union Medical College, Beijing, 100193, P.R. China

## Abstract

We previously developed a web server CPGAVAS for annotation, visualization and GenBank submission of plastome sequences. Here, we upgrade the server into CPGAVAS2 to address the following challenges: (i) inaccurate annotation in the reference sequence likely causing the propagation of errors; (ii) difficulty in the annotation of small exons of genes *petB, petD* and *rps16* and trans-splicing gene *rps12*; (iii) lack of annotation for other genome features and their visualization, such as repeat elements; and (iv) lack of modules for diversity analysis of plastomes. In particular, CPGAVAS2 provides two reference datasets for plastome annotation. The first dataset contains 43 plastomes whose annotation have been validated or corrected by RNA-seq data. The second one contains 2544 plastomes curated with sequence alignment. Two new algorithms are also implemented to correctly annotate small exons and trans-splicing genes. Tandem and dispersed repeats are identified, whose results are displayed on a circular map together with the annotated genes. DNA-seq and RNA-seq data can be uploaded for identification of single-nucleotide polymorphism sites and RNA-editing sites. The results of two case studies show that CPGAVAS2 annotates better than several other servers. CPGAVAS2 will likely become an indispensible tool for plastome research and can be accessed from http://www.herbalgenomics.org/cpgavas2.

## INTRODUCTION

Plastomes have been widely used in phylogenetic classification and evolutionary studies of plants (1). Obtaining a complete plastome sequence has become a laboratory routine given the advancement in next-generation DNA sequencing (NGS) technologies and a wide range of bioinformatic methods. By December 2018, more than 3000 plastome sequences have become available in GenBank, comparing to only 255 in 2012. The rapid generation of plastome sequences has led to a need to develop rapid and accurate plastome annotation methods. Several automated tools developed in the past years include DOGMA (2), CPGAVAS (3), MFannot (unpublished), Plann (4), AGORA (5), GeSeq (6) and Verdant (7). Plann and AGORA support user-provided dataset as reference. By contrast, DOGMA, CPGAVAS and Verdant rely on internal plastome databases. GeSeq supports the use of both internal and external references. Although these tools have been used extensively, most recent advancement in plastome research has presented new challenges and demands for such tools.

First, more than ever, high-quality reference datasets are required because they are the most important factor contributing to annotation quality. Mis-annotation will lead to inaccurate results. Even worse, this practice might result in the propagation of incorrect information in the public database, which will take much effort to correct. The availability of high-throughput sequencing data, such as RNA-seq data, has made possible the accurate determination of exon–intron boundaries. These RNA-seq data should not only be used to annotate newly sequenced plastomes but also to validate and update previous plastome annotation whenever possible. Second, annotation of the most simple structure genes (i.e. genes have one exon) is no longer challenging. Effort should focus on annotating genes with complex structures, such as multiple exons, small exons and trans-spliced exons. Third, repeat elements from plastomes have been widely used as markers for population genetics study (8). Analyses of plastomes should go beyond gene annotation. The identification of tandem and disperse repeats should be incorporated into the annotation pipeline. Fourth, the multiplicity nature of plastids in a cell makes plastomes an interesting subject to study intra-individual polymorphism. Single-nucleotide polymorphism (SNP) can be used as a powerful tool for species differentiation (9,10). Furthermore, the presence of mechanisms to generate RNA diversity, such as RNA-editing, has been reported for plastids (11). In this regard, new tools should be incorporated to explore plastome diversities at DNA and RNA levels taking advantage of NGS data, such as RNA-seq and Iso-seq data. At last, regardless of how accurate a computational pipeline is, manual curation is always needed to ensure the production of correct annotation; thus, the output of such a pipeline should be able to be imported to other tools for editing. To meet these new challenges and demands, we have upgraded the CPGAVAS server into CPGAVAS2.

## RESULTS AND DISCUSSION

### Implementation

CPGAVAS2 is built upon Perl MVC framework Catalyst v5.9. The analysis pipelines contain a set of modules implemented with perl v5.16, python v2.7.15, and Biopython v1.6.8. The pipelines also utilize several third party tools, such as Maker v2.31.10 (12), NCBI Blast+ v2.8.1 (13), drawgenemap v1.0 (14), tRNAscan-SE v2.0.2 (15). ARAGORN v1.2.36 (16), MUSCLE v3.8.31 (17), Bowtie2 v2.3.4 (18), bwa v0.7.12-r1039 (19), samtools v1.3.1 (20), tophat v2.1.1 (21), REDItools v1.0.4 (22), vmatch v2.3.0 (23), MISA v1.0 (24) and TRF v4.0.9 (25). In addition, the following software tools are used to create the 43-plastome dataset and the 2544-plastome dataset: CondonCode Aligner v7.01, MEGA X (26) and tablet v1.17.08.17 (27). The output GFF3 file is tested using Apollo editor v1.11.8 (28). CPGAVAS2 has been tested successful on modern browsers, namely, Internet explorer v11.0, Firefox v65.0 and Chrome v72.0.

### Overview

**Figure 1.**
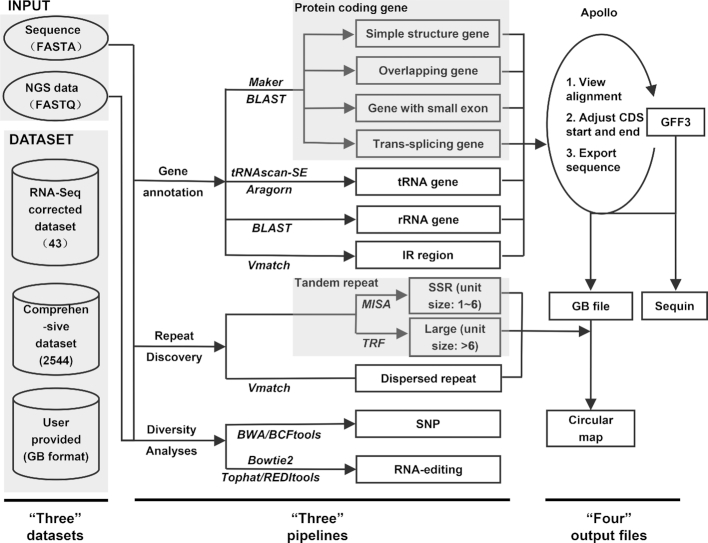
Overall CPGAVAS2 database architecture and analysis workflow. The input includes the sequence in FASTA format or NGS data in FASTQ format. Three types of dataset are shown: the expert-curated dataset, the comprehensive dataset, and the custom provided sequence. Three types of analyses are shown, namely, gene annotation, repeat identification, exploratory SNP discovery and RNA-editing site prediction. The output results include GFF3, GenBank, Sequin and a circular map files. The annotation results can be edited using third party tools such as Apollo genome editor.

CPGAVAS2 takes a plastome sequence in FASTA format and optional NGS data in FASTQ format as input. The datasets, analysis pipelines, and output files are shown in Figure 1 and summarized as three numbers: ‘three,’ ‘three,’ and ‘four.’ The first ‘three’ indicates that CPGAVAS2 supports three different datasets for annotation such as the RNA-seq data corrected dataset (the 43-plastome dataset), the comprehensive public dataset (the 2544-plastome dataset) and the user-provided sequence. The second ‘three’ indicates that CPGAVAS2 supports three types of pipeline, namely, genome annotation, repeat identification and (exploratory) diversity analysis. The third ‘four’ indicates that CPGAVAS2 produces four types of output: a GFF3 file for manual editing using editors, such as Apollo; a graphic file showing the genes and repeats annotated; a file in GenBank format; and a set of sequin file for GenBank submission.

A comparison of CPGAVAS2 and several other widely used tools are shown in Supplementary Table S1. Several major differences include the following. (i) CPGAVAS2 is the only tool supporting the use of three types of reference datasets. (ii) The 43-plastome dataset has been curated based on multiple sequence alignment and mapping of RNA-seq reads. (iii) The 43-plastome dataset covers all plastomes included in GeSeq and DOGMA at the genus level (Supplementary Table S2). (iv) CPGAVAS2 is the only web server supporting the integrated identification of repeat elements for plastomes. (v) CPGAVAS is the only web server supporting the integrated discovery of SNPs and RNA-editing sites for plastomes. Details for the datasets, analysis modules, and output files are described below.

### Reference datasets

Current annotations are abundant with uncertainty and errors, even for well-studied organisms. For example, the 5′ of *matK* genes is annotated differently for thirteen species belonging to the *Arabidopsis* genus (Supplementary Figure S1). The CDS from eleven of them is 66 bps longer than those from the other two including *Arabidopsis thaliana*. The position for the actual translation starting site remains uncertain. Second, one rRNA gene rrn5S is missing in the plastome annotation of *A. thaliana* (NC_000932.1) (Supplementary Figure S2).

**Figure 2.**
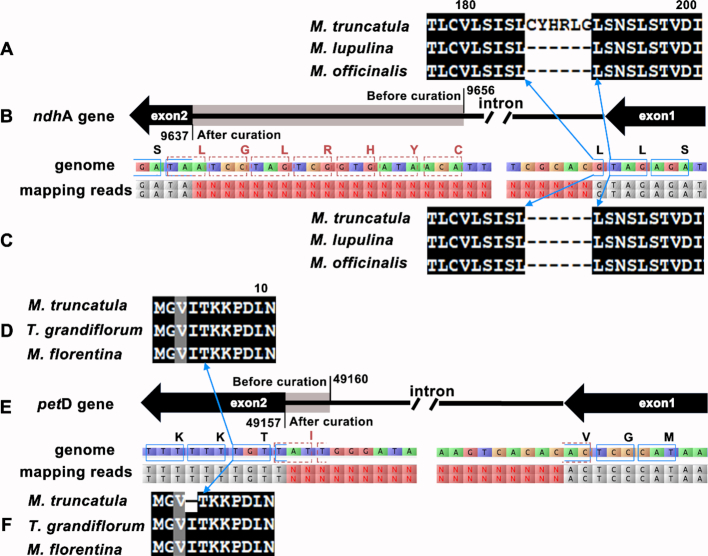
Examples for the curation of the exon–intron boundaries of genes *ndh*A and *pet*D from *Medicago truncatula* plastome (NC_003119.8) using RNA-seq data. (**A**) the alignment of the *ndh*A protein and its homologs from *Medicago lupulina* and *Melilotus officinalis* before correction. (**B**) The determination of the exon–intron boundary using RNA-seq data. A schematic representation of the exon and intron structure is shown at the top. The gene is coded on the negative strand. The dark area in the thick arrows represents the coding sequences consistent with the mapping results of RNA-seq reads. The gray area in the arrow represented coding sequences predicted computationally, which was found to be wrong based on the mapping results of RNA-seq data. The positive strand of the reference sequence and the mapping of the reads are shown at the bottom. Before correction, the exon boundary is at position 9656. After correction, the exon boundary is at position 9637. The codons ‘CYHRLG’ removed after RNA-seq data correction are shown in red and surrounded with red dashed squares. The adjacent codons are surrounded with blue solid squares. The amino acids and their codons are connected with blue line with arrow. (**C**) The alignment of the corrected ndhA protein and its homologs. (**D**) The alignment of *pet*D protein and its homologs from *Trifolium grandiflorum* and *Malus florentina*. (**E**) The mapping of the RNA-seq reads to the exon–intron boundary, which is displayed in the same way as in panel (B). The exon boundaries are at position 49 160 and 49 157 before and after correction, respectively. (**F**) the alignment of the corrected *pet*D protein and its homologs.

Another type of error is the incorrect assignment of exon–intron boundaries. Using the *ndhA* gene from *Medicago truncatula* as the first example, the alignment of the protein sequence before correction, the mapping of RNA-seq reads to the reference, and the alignment of the protein sequence after correction are shown in Figure 2A, B and C, respectively. The detailed mapping results are shown in Supplementary Figure S3A and B. As a second example, the results for *petD* genes from *M. truncatula* are shown in Figure 2D, E and F, respectively. The detailed mapping results are shown in Supplementary Figure S3C. For *ndhA*, examination of the protein alignment can provide hints for possible errors in this region. By contrast, for *petD*, examination of the protein alignment alone provides no hints for possible errors in this region. Therefore, multiple sequence alignment alone is not sufficient to find errors at the exon–intron boundaries. Mapping of RNA-seq reads to the reference is critical for identifying all exon–intron boundaries.

For this reason, we constructed a dataset containing 43 plastomes that were curated using RNA-seq data. These include all plastomes used in the datasets from GeSeq and DOGMA at genus level (Supplementary Table S2). First, we downloaded the RNA-seq data with paired reads from the NCBI SRA database (https://www.ncbi.nlm.nih.gov/sra/) for each organism. Second, the sequencing reads were mapped to the corresponding reference sequence by using tophat (v2.1.1) with the typical parameters: tophat –library-type fr-unstranded –max-intron-length 2000 –coverage-search –microexon-search. At last, the mapping results were visualized using Tablet and examined by eyeballing. In total, 60 genes were found to have incorrect exon–intron boundaries and the coding sequences (CDS) was corrected based on the mapping results. Details for the corrected CDS and protein sequences are shown in Supplementary File S1.

However, 43 plastomes only represent a small fraction of plastome sequences that are currently available and might not contain the most closely related sequence for a particular query. To overcome this limitation, the 43 plastomes were combined with another 2501 plastomes available from the public database to form a 2544-plastome dataset. The taxonomic distribution of these 2544 plastomes is shown in Supplementary Table S3. The 2501 annotations were curated by sequence similarity comparison. From the 2544 plastomes, more than 118 genes have been annotated, among them, 80 genes are the most abundant, the least abundant of which is the ycf15 gene found in 559 plastomes. These 80 protein coding genes and 4 rRNA genes are used in annotation. In the case when the user would like to use particular reference sequence, CPGAVAS2 allows the user to provide the sequence in GenBank format as reference.

### Identifying genes

The gene identification pipeline can be divided into two parts. The first part was optimized based on the pipelines implemented in CPGAVAS (3). The second part was developed in this study to identify genes (see below) that have complex structures and are challenging to annotate.

### Identifying small exons of *petB, petD* and *rpl16*

Several plastome genes contain small exons that are too short to be annotated using similarity based methods, such as BLASTN. In *A. thaliana*, three genes, namely, *petB, petD* and *rpl16*, have small exons, which are 6, 8 and 9 bp long, respectively. Previously, Verdant (7) designed an algorithm to identify these small exons. Verdant hypothesizes that the CDS of small exons are highly conserved. This tool first identifies all DNA sequences in the plastome that correctly match the conserved CDS of small exons; then it selects the match that is immediately upstream of the second exon as the CDS of the small exon. We extracted and aligned the CDS of all small exons from the 2544 plastomes. The sequence ‘ATGAGT’ is the most abundant pattern for gene *petB* with a count of 1759 (69.1%, Supplementary Figure S4A). In contrast, the sequence ‘ATGGGAGT’ is the most abundant pattern for gene *petD* with a count of 1833 (72.1%, Supplementary Figure S4B). At last, the sequence ‘ATGCTTAGT’ is the most abundant pattern for gene *rpl16* with a count of 1846 (72.6%, Supplementary Figure S4C). The underline bases represent the start codons. Since the CDS of the small exons are not 100% conserved, Verdant's method will likely miss the small exons, for which the CDS are different from the conserved consensus sequence. Furthermore, it is possible that the match immediately upstream the second exon may locate in the actual introns that might have the same pattern.

In view of these limitations, a novel method is in need to accurately annotate the small exons of these genes. Using *A. thaliana* as an example, we mapped RNA-seq reads to the plastome sequence. It suggests that the 5′ untranslated regions (UTR) of all the three genes *petB, petD* and *rpl16* are longer than 50 bp (Supplementary Figure S5A–C). To determine how conserved the 5′ UTR sequences are, the 50 bp sequences immediately upstream of the start codon of these genes from 134 family in our 2544-plastome dataset were extracted and subjected to multiple sequence alignment. As an example, the alignments of the sequences for genes *petB, petD* and *rpl16* from 79, 71 and 80 Brassicaceae plants are shown in Supplementary Figure S6A, B and C, respectively. It is observed that the 5′ UTR are highly conserved, at least at the family level.

Based on these observations, we developed an algorithm called ‘Identifying Small Exons based on Conserved 5′UTR Sequences’. The ISECUS algorithm first extracts the CDS of the small exons and the 50 bp long 5′ UTR sequences from the reference sequences. Second, these sequences are used to scan the plastome sequence to be annotated using BLASTN (*E*-value = 1e-20). From the hit sequence, ISECUS algorithm extracts the CDS from the 3′ end based on the length of small exons in the reference sequence. Our internal test showed that 50 bp long sequences are sufficiently specific to find the correct hit and ISECUS can find the CDS of the small exons rather accurately. However, this algorithm might fail in cases when the conditions for the above heuristic rules cannot be satisfied (data not shown).

### Identifying *rps12*

The trans-splicing gene *rps12* typically has two to three exons. A schematic representation of the genomic organization of *rps12* gene in *A. thaliana* is shown in Supplementary Figure S7. The first exon is located in the large single-copy region (LSC), and the second or third (if present) exons are located in the inverted repeat regions (IR). The exons of the genes were joined together to form the final two transcripts. The annotation of *rps12* presents two challenges: the last exon is too short to be identified by sequence similarity based method such as BLASTN, similar to that for small exons described above; and for a plastome having two IRs, two transcripts can be produced with one exon shared by the two transcripts (Supplementary Figure S7). Thus far, joining different exons to produce full-length transcripts automatically is difficult.

We first examined the pattern and statistics of the last exon of *rps12* from the 2544 plastomes. The 26 bp sequence ‘AATATGGGGTCAAAAAGCCAAAATAA’ is the most conserved pattern with a count of 1144 (45.0%, Supplementary Figure S8). The underline bases represent the stop codon. Then, we examine the level of conservation of the 3′ UTR sequences. The 50 bp sequences downstream of the stop codons of the *rps12* genes from 134 families included in our 2544-plastome dataset were extracted and subjected to multiple sequence alignment. As an example, the alignments of the 3′ UTR of the *rps12* genes from 75 Brassicaceae plants were shown in Supplementary Figure S9. The results show that these sequences are highly conserved, at least at the family level. Based on these observations, we applied the ISECUS algorithm to identify the short exon. In contrast to what described above for *petB, petD* and *rps16*, the conserved 3′ UTR sequence was used. To overcome the second challenge, we developed an algorithm called ‘Identify Trans-splicing Gene by Individual Exons’ (ITGIE). The exon one and two of *rps12* gene were compiled to form two separate databases. Each of them were identified with the corresponding database in the same way as those for the genes with one exon. Once all three exons were identified, they were joined together based on the two configurations observed in the reference sequence as shown in Supplementary Figure S7. Similar strategies were adopted to annotate *rps12* genes with only two exons.

### Identifying problematic predictions for tRNA genes

There are several widely used tRNA gene prediction tools, including tRNAscan-SE and ARAGORN. Using the GenBank annotation (NC_000932.1) for *A. thaliana* as an example, we compared the performance of tRNAscan-SE and ARAGORN on three aspects: (i) consistency in the prediction of tRNA genes with intron; (ii) consistency in the prediction of tRNA genes without intron; and (iii) the consistency in the naming of the predicted tRNA genes. As shown in Supplementary Table S4, tRNAscan-SE predicted the tRNA genes without intron better than ARAGORN. In contrast, ARAGORN predicted the tRNA genes with intron better than tRNAscan-SE. Both of them predicted some tRNA genes with incorrect names. Based on these observations, we implemented a corresponding tRNA prediction pipeline. In short, both tRNAscan and ARAGORN are used to predict tRNA genes initially. Those prediction results from tRNAscan-SE for genes without intron are saved, while those prediction results from ARAGORN for genes with intron are saved. These saved tRNA genes were used to search tRNAdb based on sequence similarity (29). The predicted tRNA genes whose names are not the same as their best hits are written into a warning file for expert inspection and curation.

### Discovering repeats

**Figure 3.**
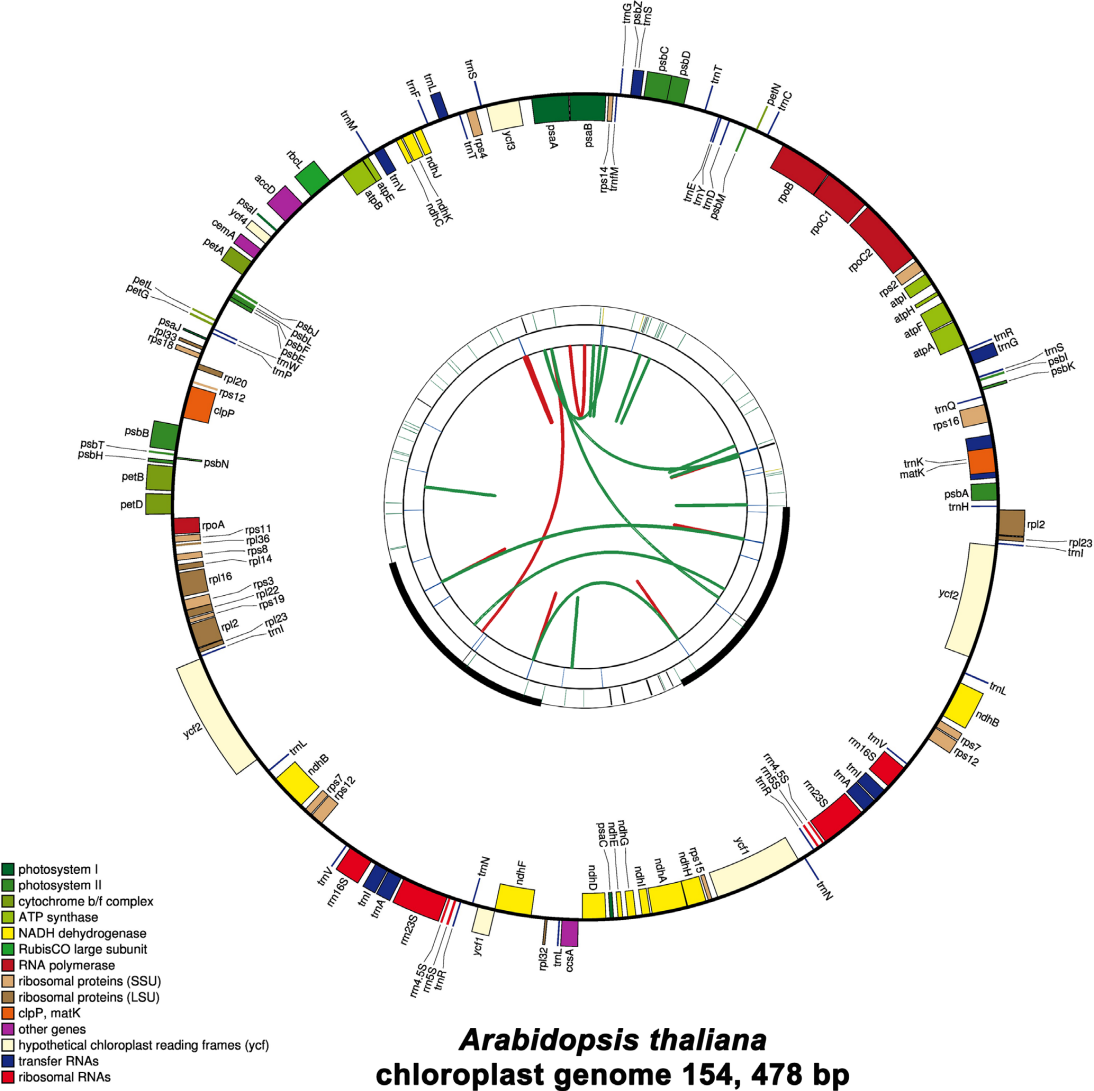
Graphic representation of features identified in the plastome of *Arabidopsis thaliana* by using CPGAVAS2. The map contains four rings. From the center going outward, the first circle shows the forward and reverse repeats connected with red and green arcs, respectively. The next circle shows the tandem repeats marked with short bars. The third circle shows the microsatellite sequences identified using MISA. The fourth circle is drawn using drawgenemap and shows the gene structure on the plastome. The genes were colored based on their functional categories, which are shown at the left corner.

Sequence repeats play important roles in diverse applications, including genetic diversity, linkage/association mapping of gene/quantitative trait loci, marker-assisted selection, variety identification and evolution analysis (30). Simple sequence repeats (SSRs), including those from plastomes, have been widely used as genetic markers to distinguish individuals and species. We adopted three widely used tools to identify tandem repeats and dispersed repeats. The tandem repeats are divided into two types. The first type is also called SSR and has repeat unit size ranging from 1 to 6 bp. The second type is called longer tandem repeat and has repeat unit size >6 bp. As shown in Figure 1, the repeat discovery pipeline calls three tools. In particular, MISA is used to discover SSRs, TRF is used to discovery longer tandem repeats, and vmatch is used to discover dispersed repeats. The results were incorporated into the output circular map for visualization (Figure 3). This pipeline allows rapid repeat discovery for bench biologists. It should be pointed out that these tools use different algorithms to discover repeats. In addition, there are complex repeats that might contain multiple types of simple repeat overlapping with each other. Expert examination is needed to determine whether or not to use these repeats for their particular purposes. Details regarding the input format, output format, and parameters of this pipeline can be found in our online manual.

### Analyzing diversity

We previously found multiple heteroplasmic regions in the plastome of *Astragalus membranaceus*. Several heteroplasmic regions are highly variable and can be used as polymorphic markers for evolutionary and classification studies at cellular, individual and lower taxonomic levels (31). To allow rapid identification of these highly polymorphic regions, we implemented a module for SNP identification by using NGS data related to the plastome to be annotated. Various methods used for plastome SNP discovery from NGS data were compared previously (10). Basing on the results, we select BWA and BCFtools for SNP discovery because these programs are more lightweight and more versatile compared with other programs.

RNA-editing is the process of modifying mRNA to generate diverse types of proteins from the same genomic sequences (32). Identification of RNA-editing sites will extend our understanding regarding the restoring evolutionary conserved amino acids in plastids (33). Although several RNA-editing site tools have been developed, to our knowledge, no web server is available (34,35). Here, we incorporated a RNA editing site analysis (REA) pipeline we used before into CPGAVAS2 (36). To further evaluate its performance, a RNA-seq dataset (SRR1004790) for *A. thaliana* was analyzed using REA. As shown in Supplementary File S2, a total of 171 C-T/A-G editing sites were identified. Among them, 35 was consistent with 34 major and 1 minor RNA editing sites out of 43 sites previously identified (37). The remaining eight minor RNA editing sites of the 43 known sites were not identified, probably due to the low sequence coverage or the inappropriate plant samples used for the study, in which these sites might not have been edited efficiently.

To use these modules, users need to upload their NGS data to the web server, which might be a problem for a large dataset. To overcome this difficulty, users are recommended to perform preliminary filtering of the NGS reads instead of directly loading the entire raw NGS reads to the server. We provided a pre-processing tool (PREA) for the users to enrich reads for the discovery of RNA editing sites. Essentially, RNA-seq reads were filtered for particular set of genes by using sequence similarity based comparison tools such as BLASTN. As an example, we used PREA to enrich reads for *ndhB* genes. This gene has a total of twelve known RNA editing sites, the largest number among chloroplast genes. As shown in Supplementary File S3, a total of nine C-T/A-G editing sites were identified with the coverage from 130–4409 on the sense strand, matching nine of the twelve known sites. At last, source codes for these modules have been released to github. Interested users can install the scripts locally and run the analyses in their own environment.

## EVALUATION

**Figure 4.**
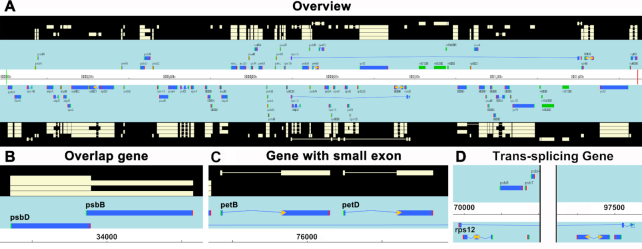
Representative analysis results of CPGAVAS2 viewed with Apollo genome editor. (A) overview of plastome annotations. The picture can be divided into three areas. The area with black background display various features mapped to the genome sequence, which is shown in the area with white background as a line. The numbers on the line represent the base positions. The area with light blue background shows the predicted genes aligned to the genome sequence. Exons represented by thick blue lines are connected with thin lines representing the introns. The genes above the genome sequence are coded on the positive strand, while those below the genome sequence are coded on the negative strand. (B) The correct identification of overlapping genes *psb*D and *psb*B. (C) The correct identification of the small exons for *pet*B and *pet*D, which are 6 and 8 bp long, respectively. (D) The correct identification of *rps*12, who has one exon in the LSC region and one or more exons in the IR regions.

CPGAVAS2 generates a circular map displaying the annotated genes and the identified repeats in the plastome (Figure 3). The outer ring is generated with the popular drawgenemap script from GeSeq. The inner three rings show the SSR, long tandem and dispersed repeats identified using CPGAVAS2. The annotation results are best visualized in Apollo genome editor, and an example is shown in Figure 4. Panel A shows the overall annotation results. Panels B, C, and D show the correct identification of overlapping genes, small exons and *rps12* by CPGAVAS2, respectively. To evaluate the performance of CPGAVAS2, we select two plastome sequences as test data. We initially tried to compare the performance of six tools: AGORA, GeSeq, Verdant, MFannot, DOGMA and CPGAVAS2. As Verdant did not respond to sequence submission after multiple attempts, we only compared the other five tools.

### Case study 1

*Arabidopsis thaliana* was used as the first test sequence because it has been well studied with correct annotation. We used user-provided sequence as reference for annotation whenever possible. The detailed comparison results are shown in Supplementary File S4, and a summary of the results is shown in Supplementary Table S5. GeSeq and MFannot annotated one and three genes, respectively, with incorrect names. Most errors were found when predicting the start and end positions of genes. About two, eleven, five, eleven and thirteen genes had incorrect start or end positions predicted by CPGVAS2, AGORA, GeSeq, MFannot and DOGMA, respectively. In addition, AGORA, GeSeq, MFannot and DOGMA failed to predict some exons of nine, three, five and six genes, respectively. For three genes having small exons, only CPGAVAS2 correctly identified all of them. For the trans-splicing gene, CPGAVAS2 predicted the three exons correctly and joined them together. GeSeq and DOGMA predicted all three exons but failed to join them together. MFannot predicted two exons and joined them together. For rRNA gene prediction, the current gold standard annotation from GenBank has one rrn5S gene missing, and MFannot failed to predict the rrn4.5S gene (Supplementary Table S5).

### Case study 

We then used a newly sequenced plastome from *Glechomalongituba* as test sequence. The plastome is not yet available in public database. RNA-seq data are available (SRX2468822) and are used to construct a ‘true’ annotation for comparison. The FASTA sequence of the plastome is provided in Supplementary File S5. The detailed comparison results are shown in Supplementary File S6, and a summary of the results is shown in Supplementary Table S6. MFannot and DOGMA predicted seven and six genes with incorrect names. GeSeq and DOGMA failed to predict one and five genes, respectively. AGORA, GeSeq, MFannot and DOGMA failed to predict some exons of eight, three, five and five genes, respectively. Most errors were found when predicting the start and end of genes, similar to those found in case study 1. About 5, 21, 18, 20 and 5 genes had incorrect start or end positions predicted by CAPGVAS2, AGORA, GeSeq, MFannot and DOGMA, respectively. For three genes with small exons, only CPGAVAS2 correctly identified all of them. The performance of predicting trans-splicing gene *rps12* is similar to those shown in case study 1, with CPGAVAS2 predicting all three exons and joining them together correctly.

## CONCLUSION

We have upgraded our previous web server CPGAVAS into CPGAVAS2, with the addition of new functions. We constructed two datasets. The 43-plastome dataset is curated with RNA-seq data, and the 2544-plastome dataset contains the largest number of plastome sequences among similar tools. CPGAVAS2 accepts user-provided reference sequence. In addition to predicting structurally simple genes, we developed two algorithms to annotate structurally complex genes such as those having small exons or trans-splicing exons. In addition, CPGAVAS2 discovers repeats automatically after annotating the plastome, whose results are presented in a circular map together with the annotation of genes. At last, CPGAVAS2 supports the exploratory analyses of plastome diversity by identifying SNPs and RNA-editing sites if user supplies the NGS data. We believe CPGAVAS2 will become a powerful tool for plastome research in the NGS era.

## Supplementary Material

gkz345_Supplemental_Files
