# Supplementary material for: CPGAVAS2, an integrated plastome sequence annotator and analyzer
Source: Nucleic Acids Res. 2019 May 8;47(W1):W65–73. doi: 10.1093/nar/gkz345 (PMC6602467; doi:10.1093/nar/gkz345)
Supplement: gkz345_Supplemental_Files [file gkz345_supplemental_files.zip › 201904151100-CPGAVAS2-suppl.docx]

### SUPPLEMENTARY MATERIALS

###### Total counts:

Supplementary tables: 6

Supplementary figures: 9

Supplementary files: 6

###### Table S1 Comparison of the general features of CPGAVAS2 and four other web servers

| **Features or Functions** |  | **Name of Web Server** | | | | |  |
| --- | --- | --- | --- | --- | --- | --- | --- |
|  | **CPGAVAS2** | | **AGORA** | **GeSeq** | **DOGMA** | **MFannot** | |
| Types of reference dataset supported | 1. 43-plastome dataset validated with RNA-seq data,  2. 2544-plastome dataset curated with sequence comparison,  3. User provided dataset | | User provided dataset | 1. Curated dataset, 2. User provided dataset | Curated dataset | Unknown | |
| Method for creating curated dataset | Sequence alignment, RNA-seq data mapping | | no | Sequence alignment | Sequence alignment | Unknown | |
| Support User-provided reference | yes | | yes | Yes | no | no | |
| No of plastome in the comprehensive dataset | 2544 | | 0 | 0 | 0 | Unknown | |
| No. of plastome in the curated dataset | 43 | | 0 | 34 | 16 | Unknown | |
| Repeat discovery | yes | | no | no | no | no | |
| Diversity analysis (Exploratory) | yes | | no | no | no | no | |
| Output format | GFF3, GenBank, FASTA, Sequin | | CSV, GenBank, Fasta, PDF, Zipped | GenBank | Plain text | Feature table, Sequin | |
| Visualization of results | yes | | yes | yes | yes | No | |

###### Table S2 List of plastomes included in the RNA-seq data curated dataset.

The meanings of column are: the species name, the accession numbers of the corresponding plastomes, the taxonomy lineage of the species, the accession number for the corresponding RNA-seq data. "N/A": not assigned at this taxonomic rank.

| **Species** | **NCBI RefSeq** | **Phylum** | **Class** | **Order** | **Family** | **Genus** | **SRA Accession** | **Included in** | |
| --- | --- | --- | --- | --- | --- | --- | --- | --- | --- |
|  |  |  |  |  |  |  |  | **GeSeq** | **DOGMA** |
| *Atropa belladonna* | NC_004561.1 | Streptophyta | N/A | Solales | Solaceae | Atropa | SRX060269 | yes | no |
| *Adiantum shastense* | NC_037478.1 | Streptophyta | Polypodiopsida | Polypodiales | Pteridaceae | Adiantum | SRX3362765 | yes | yes |
| *Anthoceros formosae* | NC_004543.1 | Streptophyta | Anthocerotopsida | Anthocerotales | Anthocerotaceae | Anthoceros | SRX3066668 | yes | no |
| *Arabidopsis thaliana* | NC_000932.1 | Streptophyta | N/A | Brassicales | Brassicaceae | Arabidopsis | SRR1004790 | yes | yes |
| *Brachypodium distachyon* | NC_011032.1 | Streptophyta | Liliopsida | Poales | Poaceae | Brachypodium | SRX3748523 | yes | no |
| *Elaeis guineensis* | NC_017602.1 | Streptophyta | Liliopsida | Arecales | Arecaceae | Elaeis | SRX4663593 | yes | no |
| *Ginkgo biloba* | NC_016986.1 | Streptophyta | N/A | Ginkgoales | Ginkgoaceae | Ginkgo | SRX3633931 | yes | no |
| *Glycine max* | NC_007942.1 | Streptophyta | N/A | Fabales | Fabaceae | Glycine | SRX4120937, SRX3646145 | yes | no |
| *Hevea brasiliensis* | NC_015308.1 | Streptophyta | N/A | Malpighiales | Euphorbiaceae | Hevea | SRX5181862 | yes | no |
| *Hordeum vulgare* | NC_008590.1 | Streptophyta | Liliopsida | Poales | Poaceae | Hordeum | SRX4095427 | yes | no |
| *Lotus japonicus* | NC_002694.1 | Streptophyta | N/A | Fabales | Fabaceae | Lotus | DRX001774 | yes | yes |
| *Marchantia polymorpha* subsp. *Ruderalis* | NC_037507.1 | Streptophyta | Marchantiopsida | Marchantiales | Marchantiaceae | Marchantia | DRX123504 | yes | yes |
| *Medicago truncatula* | NC_003119.8 | Streptophyta | N/A | Fabales | Fabaceae | Medicago | SRX658552, SRR7738223 | yes | no |
| *Nicotiana tabacum* | NC_001879.2 | Streptophyta | N/A | Solales | Solaceae | Nicotia | SRX4941542 | yes | yes |
| *Oenothera elata* ssp. *hookeri* | NC_002693.2 | Streptophyta | N/A | Myrtales | Ograceae | Oenothera | ERR706819 | yes | yes |
| *Oryza sativa* ssp. *japonica* | NC_001320.1 | Streptophyta | Liliopsida | Poales | Poaceae | Oryza | SRR1618549 | yes | yes |
| *Phalaenopsis aphrodite* | NC_007499.1 | Streptophyta | Liliopsida | Asparagales | Orchidaceae | Phalaenopsis | SRX2439762 | yes | no |
| *Pisum sativum* | NC_014057.1 | Streptophyta | N/A | Fabales | Fabaceae | Pisum | SRX952515 | yes | no |
| *Pinus bungeana* | NC_028421.1 | Streptophyta | N/A | Piles | Piceae | Pinus | SRR4033079 | yes | yes |
| *Populus trichocarpa* | NC_009143.1 | Streptophyta | N/A | Malpighiales | Salicaceae | Populus | SRX4137198 | yes | no |
| *Ricinus communis* | NC_016736.1 | Streptophyta | N/A | Malpighiales | Euphorbiaceae | Ricinus | ERX021378 | yes | no |
| *Sorghum bicolor* | NC_008602.1 | Streptophyta | Liliopsida | Poales | Poaceae | Sorghum | SRX5210763 | yes | no |
| *Solanum lycopersicum* | NC_007898.3 | Streptophyta | N/A | Solales | Solaceae | Solanum | SRX3766995 | yes | no |
| *Selaginella moellendorffii* | NC_013086.1 | Streptophyta | Lycopodiopsida | Selaginellales | Selaginellaceae | Selaginella | SRX2312496 | yes | no |
| *Spinacia oleracea* | NC_002202.1 | Streptophyta | N/A | Caryophyllales | Chenopodiaceae | Spicia | DRR057923 | yes | yes |
| *Solanum tuberosum* | NC_008096.2 | Streptophyta | N/A | Solales | Solaceae | Solanum | SRX4415371, SRX4415387 | yes | no |
| *Vitis vinifera* | NC_007957.1 | Streptophyta | N/A | Vitales | Vitaceae | Vitis | SRX3680594 | yes | no |
| *Zea mays* | NC_001666.2 | Streptophyta | Liliopsida | Poales | Poaceae | Zea | SRR7903797 | yes | yes |
| *Chlorella vulgaris* | NC_001865.1 | Chlorophyta | Trebouxiophyceae | Chlorellales | Chlorellaceae | Chlorella | SRX2581307 | no | yes |
| *Epifagus virginiana* | NC_001568.1 | Streptophyta | N/A | Lamiales | Orobanchaceae | Epifagus | ERX2099648 | no | yes |
| *Mesostigma viride* | NC_002186.1 | Streptophyta | Mesostigmatophyceae | Mesostigmatales | Mesostigmataceae | Mesostigma | SRX718287 | no | yes |
| *Nephroselmis olivacea* | NC_000927.1 | Chlorophyta | Nephroselmidophyceae | N/A | N/A | Nephroselmis | ERX2100062 | no | yes |
| *Psilotum nudum* | NC_003386.1 | Streptophyta | Polypodiopsida | Psilotales | Psilotaceae | Psilotum | SRX1098247 | no | yes |
| *Triticum aestivum* | NC_002762.1 | Streptophyta | Liliopsida | Poales | Poaceae | Triticum | ERX2922895 | no | yes |
| *Salvia miltiorrhiza* | NC_020431.1 | Streptophyta | N/A | Lamiales | Lamiaceae | Salvia | SRX388784 | no | no |
| *Astragalus membranaceus* | NC_029828.1 | Streptophyta | N/A | Fabales | Fabaceae | Astragalus | ERX651043 | no | no |
| *Trieres chinensis* | NC_001713.1 | Bacillariophyta | Mediophyceae | Triceratiales | Triceratiaceae | Trieres | SRX551289 | no | no |
| *Panax ginseng* | NC_006290.1 | Streptophyta | N/A | Apiales | Araliaceae | Pax | SRX758350 | no | no |
| *Huperzia lucidula* | NC_006861.1 | Streptophyta | Lycopodiopsida | Lycopodiales | Lycopodiaceae | Huperzia | ERX2099921 | no | no |
| *Saccharum officinarum* | NC_006084.1 | Streptophyta | Liliopsida | Poales | Poaceae | Saccharum | SRX4157295 | no | no |
| *Brassica nigra* | NC_030450.1 | Streptophyta | N/A | Brassicales | Brassicaceae | Brassica | ERX2099492 | no | no |
| *Cuscuta reflexa* | NC_009766.1 | Streptophyta | N/A | Solales | Convolvulaceae | Cuscuta | SRX472174 | no | no |
| *Magnolia grandiflora* | NC_020318.1 | Streptophyta | N/A | Magnoliales | Magnoliaceae | Magnolia | ERX2099196 | no | no |
| *Glechoma longituha* | 20170403 | Streptophyta | N/A | Lamiales | Lamiaceae | *Glechoma* | SRX2468822 | no | no |

###### Table S3 The taxonomy classification at the family level for plastome sequences in the comprehensive dataset. "N/A": not assigned at this rank.

| **Phylum** | **Class** | **Order** | **Family** | **No. of Genome** |
| --- | --- | --- | --- | --- |
| Streptophyta | N/A | Lamiales | Acanthaceae | 6 |
| Streptophyta | Liliopsida | Acorales | Acoraceae | 1 |
| Streptophyta | N/A | Ericales | Actinidiaceae | 8 |
| Streptophyta | N/A | Dipsacales | Adoxaceae | 6 |
| Streptophyta | N/A | Caryophyllales | Aizoaceae | 2 |
| Streptophyta | N/A | Brassicales | Akaniaceae | 1 |
| Streptophyta | Liliopsida | Liliales | Alstroemeriaceae | 1 |
| Streptophyta | N/A | Saxifragales | Altingiaceae | 1 |
| Streptophyta | N/A | Caryophyllales | Amaranthaceae | 2 |
| Streptophyta | Liliopsida | Asparagales | Amaryllidaceae | 9 |
| Streptophyta | Bryopsida | Hypnales | Amblystegiaceae | 1 |
| Streptophyta | N/A | Amborellales | Amborellaceae | 1 |
| Streptophyta | N/A | Santalales | Amphorogynaceae | 1 |
| Streptophyta | N/A | Sapindales | Anacardiaceae | 7 |
| Streptophyta | Jungermanniopsida | Metzgeriales | Aneuraceae | 2 |
| Streptophyta | Anthocerotopsida | Anthocerotales | Anthocerotaceae | 1 |
| Streptophyta | N/A | Apiales | Apiaceae | 32 |
| Streptophyta | N/A | Gentianales | Apocynaceae | 6 |
| Streptophyta | Liliopsida | Alismatales | Araceae | 11 |
| Streptophyta | N/A | Apiales | Araliaceae | 19 |
| Streptophyta | N/A | Araucariales | Araucariaceae | 2 |
| Streptophyta | Liliopsida | Arecales | Arecaceae | 7 |
| Streptophyta | N/A | Piperales | Aristolochiaceae | 5 |
| Streptophyta | Liliopsida | Asparagales | Asparagaceae | 34 |
| Streptophyta | Liliopsida | Asparagales | Asphodelaceae | 3 |
| Streptophyta | Polypodiopsida | Polypodiales | Aspleniaceae | 3 |
| Streptophyta | N/A | Asterales | Asteraceae | 148 |
| Streptophyta | Polypodiopsida | Polypodiales | Athyriaceae | 12 |
| Streptophyta | N/A | Ericales | Balsaminaceae | 2 |
| Chlorophyta | Mamiellophyceae | Mamiellales | Bathycoccaceae | 2 |
| Streptophyta | N/A | Ranunculales | Berberidaceae | 26 |
| Streptophyta | N/A | Fagales | Betulaceae | 46 |
| Streptophyta | N/A | Lamiales | Bignoniaceae | 26 |
| Streptophyta | Polypodiopsida | Polypodiales | Blechnaceae | 2 |
| Chlorophyta | Chlorophyceae | Sphaeropleales | Bracteacoccaceae | 3 |
| Streptophyta | N/A | Brassicales | Brassicaceae | 85 |
| Streptophyta | Liliopsida | Poales | Bromeliaceae | 1 |
| Chlorophyta | Ulvophyceae | Bryopsidales | Bryopsidaceae | 4 |
| Streptophyta | Liliopsida | Dioscoreales | Burmanniaceae | 6 |
| Streptophyta | N/A | Sapindales | Burseraceae | 2 |
| Streptophyta | N/A | Buxales | Buxaceae | 2 |
| Streptophyta | N/A | Nymphaeales | Cabombaceae | 4 |
| Streptophyta | N/A | Caryophyllales | Cactaceae | 1 |
| Streptophyta | N/A | Laurales | Calycanthaceae | 2 |
| Streptophyta | N/A | Asterales | Campanulaceae | 29 |
| Streptophyta | Liliopsida | Liliales | Campynemataceae | 1 |
| Streptophyta | N/A | Rosales | Cannabaceae | 11 |
| Streptophyta | N/A | Dipsacales | Caprifoliaceae | 15 |
| Streptophyta | N/A | Brassicales | Caricaceae | 1 |
| Streptophyta | N/A | Caryophyllales | Caryophyllaceae | 16 |
| Chlorophyta | Ulvophyceae | Bryopsidales | Caulerpaceae | 5 |
| Streptophyta | N/A | Celastrales | Celastraceae | 3 |
| Streptophyta | N/A | Ceratophyllales | Ceratophyllaceae | 1 |
| Streptophyta | N/A | Saxifragales | Cercidiphyllaceae | 1 |
| Chlorophyta | Chlorophyceae | Chaetopeltidales | Chaetopeltidaceae | 1 |
| Streptophyta | Coleochaetophyceae | Coleochaetales | Chaetosphaeridiaceae | 1 |
| Streptophyta | Charophyceae | Charales | Characeae | 1 |
| Chlorophyta | Chlorophyceae | Chlamydomonadales | Characiochloridaceae | 1 |
| Streptophyta | N/A | Caryophyllales | Chenopodiaceae | 9 |
| Chlorophyta | Chlorophyceae | Chlamydomonadales | Chlamydomonadaceae | 2 |
| Streptophyta | N/A | Chloranthales | Chloranthaceae | 4 |
| Chlorophyta | Trebouxiophyceae | Chlorellales | Chlorellaceae | 7 |
| Streptophyta | Chlorokybophyceae | Chlorokybales | Chlorokybaceae | 1 |
| Chlorophyta | Chlorophyceae | Sphaeropleales | Chromochloridaceae | 1 |
| Streptophyta | N/A | Malpighiales | Chrysobalanaceae | 50 |
| Streptophyta | Polypodiopsida | Cyatheales | Cibotiaceae | 1 |
| Streptophyta | N/A | Ranunculales | Circaeasteraceae | 2 |
| Streptophyta | N/A | Brassicales | Cleomaceae | 1 |
| Streptophyta | Zygnemophyceae | Desmidiales | Closteriaceae | 1 |
| Streptophyta | N/A | Malpighiales | Clusiaceae | 1 |
| Chlorophyta | Ulvophyceae | Bryopsidales | Codiaceae | 3 |
| Streptophyta | Liliopsida | Liliales | Colchicaceae | 2 |
| Streptophyta | Coleochaetophyceae | Coleochaetales | Coleochaetaceae | 1 |
| Streptophyta | N/A | Myrtales | Combretaceae | 1 |
| Streptophyta | N/A | Solanales | Convolvulaceae | 12 |
| Streptophyta | N/A | Cornales | Cornaceae | 1 |
| Streptophyta | N/A | Cucurbitales | Corynocarpaceae | 1 |
| Streptophyta | N/A | Saxifragales | Crassulaceae | 4 |
| Streptophyta | N/A | Cucurbitales | Cucurbitaceae | 14 |
| Streptophyta | N/A | Cupressales | Cupressaceae | 23 |
| Streptophyta | Polypodiopsida | Cyatheales | Cyatheaceae | 2 |
| Streptophyta | N/A | Cycadales | Cycadaceae | 4 |
| Streptophyta | Liliopsida | Pandanales | Cyclanthaceae | 1 |
| Streptophyta | Liliopsida | Poales | Cyperaceae | 2 |
| Streptophyta | Polypodiopsida | Polypodiales | Cystopteridaceae | 1 |
| Streptophyta | N/A | Malvales | Cytinaceae | 1 |
| Streptophyta | N/A | Saxifragales | Daphniphyllaceae | 1 |
| Streptophyta | Polypodiopsida | Polypodiales | Dennstaedtiaceae | 1 |
| Chlorophyta | Ulvophyceae | Bryopsidales | Derbesiaceae | 2 |
| Streptophyta | Zygnemophyceae | Desmidiales | Desmidiaceae | 2 |
| Streptophyta | Liliopsida | Dioscoreales | Dioscoreaceae | 21 |
| Streptophyta | Polypodiopsida | Polypodiales | Diplaziopsidaceae | 3 |
| Streptophyta | N/A | Caryophyllales | Droseraceae | 4 |
| Streptophyta | Polypodiopsida | Polypodiales | Dryopteridaceae | 4 |
| Streptophyta | Marchantiopsida | Marchantiales | Dumortieraceae | 1 |
| Chlorophyta | Chlorophyceae | Chlamydomonadales | Dunaliellaceae | 2 |
| Streptophyta | N/A | Ericales | Ebenaceae | 8 |
| Streptophyta | N/A | Rosales | Elaeagnaceae | 3 |
| Streptophyta | N/A | Ephedrales | Ephedraceae | 2 |
| Streptophyta | Polypodiopsida | Equisetales | Equisetaceae | 2 |
| Streptophyta | N/A | Ericales | Ericaceae | 1 |
| Streptophyta | N/A | Santalales | Erythropalaceae | 1 |
| Streptophyta | N/A | Malpighiales | Erythroxylaceae | 1 |
| Streptophyta | N/A | Garryales | Eucommiaceae | 1 |
| Streptophyta | N/A | Malpighiales | Euphorbiaceae | 7 |
| Streptophyta | N/A | Ranunculales | Eupteleaceae | 1 |
| Streptophyta | N/A | Fabales | Fabaceae | 106 |
| Streptophyta | N/A | Fagales | Fagaceae | 21 |
| Streptophyta | N/A | Geraniales | Francoaceae | 1 |
| Streptophyta | Bryopsida | Funariales | Funariaceae | 2 |
| Streptophyta | N/A | Gentianales | Gentianaceae | 15 |
| Streptophyta | N/A | Geraniales | Geraniaceae | 25 |
| Streptophyta | N/A | Lamiales | Gesneriaceae | 8 |
| Streptophyta | N/A | Ginkgoales | Ginkgoaceae | 1 |
| Streptophyta | Polypodiopsida | Gleicheniales | Gleicheniaceae | 1 |
| Streptophyta | N/A | Gnetales | Gnetaceae | 3 |
| Streptophyta | Jungermanniopsida | Jungermanniales | Gymnomitriaceae | 1 |
| Chlorophyta | Chlorophyceae | Chlamydomonadales | Haematococcaceae | 1 |
| Streptophyta | Liliopsida | Commelinales | Haemodoraceae | 1 |
| Streptophyta | N/A | Saxifragales | Haloragaceae | 1 |
| Streptophyta | N/A | Saxifragales | Hamamelidaceae | 5 |
| Streptophyta | N/A | Aquifoliales | Helwingiaceae | 1 |
| Streptophyta | Liliopsida | Asparagales | Hyacinthaceae | 3 |
| Streptophyta | N/A | Nymphaeales | Hydatellaceae | 1 |
| Streptophyta | N/A | Cornales | Hydrangeaceae | 4 |
| Streptophyta | Liliopsida | Alismatales | Hydrocharitaceae | 2 |
| Chlorophyta | Chlorophyceae | Sphaeropleales | Hydrodictyaceae | 8 |
| Streptophyta | Polypodiopsida | Hymenophyllales | Hymenophyllaceae | 1 |
| Streptophyta | Polypodiopsida | Polypodiales | Hypodematiaceae | 1 |
| Streptophyta | N/A | Icacinales | Icacinaceae | 1 |
| Streptophyta | Liliopsida | Asparagales | Iridaceae | 1 |
| Streptophyta | Lycopodiopsida | Isoetales | Isoetaceae | 10 |
| Streptophyta | N/A | Saxifragales | Iteaceae | 1 |
| Streptophyta | Liliopsida | Poales | Joinvilleaceae | 1 |
| Streptophyta | N/A | Fagales | Juglandaceae | 11 |
| Streptophyta | Klebsormidiophyceae | Klebsormidiales | Klebsormidiaceae | 2 |
| Chlorophyta | Trebouxiophyceae | Prasiolales | Koliellaceae | 2 |
| Streptophyta | N/A | Lamiales | Lamiaceae | 31 |
| Streptophyta | N/A | Ranunculales | Lardizabalaceae | 4 |
| Streptophyta | N/A | Laurales | Lauraceae | 22 |
| Streptophyta | N/A | Ericales | Lecythidaceae | 1 |
| Streptophyta | Leiosporocerotopsida | Leiosporocerotales | Leiosporocerotaceae | 1 |
| Streptophyta | N/A | Boraginales | Lennoaceae | 2 |
| Streptophyta | N/A | Lamiales | Lentibulariaceae | 11 |
| Streptophyta | Liliopsida | Liliales | Liliaceae | 41 |
| Streptophyta | N/A | Malpighiales | Linaceae | 1 |
| Streptophyta | N/A | Santalales | Loranthaceae | 4 |
| Streptophyta | Lycopodiopsida | Lycopodiales | Lycopodiaceae | 2 |
| Streptophyta | Polypodiopsida | Schizaeales | Lygodiaceae | 2 |
| Streptophyta | N/A | Myrtales | Lythraceae | 10 |
| Streptophyta | N/A | Magnoliales | Magnoliaceae | 26 |
| Streptophyta | N/A | Malpighiales | Malpighiaceae | 2 |
| Streptophyta | N/A | Malvales | Malvaceae | 44 |
| Chlorophyta | Mamiellophyceae | Mamiellales | Mamiellaceae | 1 |
| Streptophyta | Polypodiopsida | Marattiales | Marattiaceae | 1 |
| Streptophyta | Marchantiopsida | Marchantiales | Marchantiaceae | 2 |
| Streptophyta | Polypodiopsida | Salviniales | Marsileaceae | 1 |
| Streptophyta | Liliopsida | Liliales | Melanthiaceae | 15 |
| Streptophyta | N/A | Myrtales | Melastomataceae | 19 |
| Streptophyta | N/A | Sapindales | Meliaceae | 10 |
| Streptophyta | N/A | Ranunculales | Menispermaceae | 1 |
| Streptophyta | Mesostigmatophyceae | Mesostigmatales | Mesostigmataceae | 1 |
| Streptophyta | Zygnemophyceae | Zygnematales | Mesotaeniaceae | 4 |
| Chlorophyta | Mamiellophyceae | Monomastigales | Monomastigaceae | 1 |
| Streptophyta | N/A | Caryophyllales | Montiaceae | 1 |
| Streptophyta | N/A | Rosales | Moraceae | 7 |
| Streptophyta | Liliopsida | Zingiberales | Musaceae | 3 |
| Chlorophyta | Chlorophyceae | Sphaeropleales | Mychonastaceae | 2 |
| Streptophyta | N/A | Fagales | Myricaceae | 1 |
| Streptophyta | N/A | Myrtales | Myrtaceae | 46 |
| Chlorophyta | Chlorophyceae | Chaetophorales | N/A | 1 |
| Chlorophyta | Chlorophyceae | Chlamydomonadales | N/A | 1 |
| Chlorophyta | Trebouxiophyceae | Chlorellales | N/A | 1 |
| Chlorophyta | Ulvophyceae | N/A | N/A | 1 |
| Chlorophyta | Ulvophyceae | Oltmansiellopsidales | N/A | 1 |
| Chlorophyta | Ulvophyceae | Ignatiales | N/A | 2 |
| Chlorophyta | Trebouxiophyceae | Microthamniales | N/A | 2 |
| Chlorophyta | Nephroselmidophyceae | N/A | N/A | 2 |
| Chlorophyta | N/A | N/A | N/A | 2 |
| Chlorophyta | Chlorophyceae | Oedogoniales | N/A | 2 |
| Chlorophyta | N/A | Pyramimonadales | N/A | 2 |
| Chlorophyta | Chlorophyceae | N/A | N/A | 4 |
| Chlorophyta | Ulvophyceae | Ulotrichales | N/A | 5 |
| Chlorophyta | Trebouxiophyceae | Microthamniales | N/A | 11 |
| Streptophyta | Liliopsida | Dioscoreales | Nartheciaceae | 3 |
| Streptophyta | N/A | Proteales | Nelumbonaceae | 2 |
| Chlorophyta | Chlorophyceae | Sphaeropleales | Neochloridaceae | 2 |
| Streptophyta | N/A | Nymphaeales | Nymphaeaceae | 13 |
| Streptophyta | N/A | Cornales | Nyssaceae | 3 |
| Streptophyta | N/A | Lamiales | Oleaceae | 19 |
| Streptophyta | N/A | Myrtales | Onagraceae | 10 |
| Streptophyta | Polypodiopsida | Polypodiales | Onocleaceae | 2 |
| Chlorophyta | Trebouxiophyceae | Chlorellales | Oocystaceae | 1 |
| Streptophyta | Polypodiopsida | Ophioglossales | Ophioglossaceae | 2 |
| Streptophyta | Liliopsida | Asparagales | Orchidaceae | 103 |
| Streptophyta | N/A | Lamiales | Orobanchaceae | 25 |
| Streptophyta | Bryopsida | Orthotrichales | Orthotrichaceae | 2 |
| Streptophyta | Polypodiopsida | Osmundales | Osmundaceae | 1 |
| Streptophyta | N/A | Oxalidales | Oxalidaceae | 1 |
| Streptophyta | N/A | Saxifragales | Paeoniaceae | 9 |
| Chlorophyta | Palmophyllophyceae | Palmophyllales | Palmophyllaceae | 2 |
| Streptophyta | N/A | Ranunculales | Papaveraceae | 6 |
| Streptophyta | N/A | Malpighiales | Passifloraceae | 15 |
| Streptophyta | N/A | Lamiales | Paulowniaceae | 2 |
| Streptophyta | N/A | Lamiales | Pedaliaceae | 1 |
| Chlorophyta | Pedinophyceae | Pedinomonadales | Pedinomonadaceae | 2 |
| Streptophyta | Jungermanniopsida | Pelliales | Pelliaceae | 1 |
| Streptophyta | N/A | Ericales | Pentaphylacaceae | 1 |
| Streptophyta | N/A | Saxifragales | Penthoraceae | 1 |
| Chlorophyta | Chlorophyceae | Chlamydomonadales | Phacotaceae | 1 |
| Streptophyta | N/A | Lamiales | Phrymaceae | 3 |
| Streptophyta | N/A | Pinales | Pinaceae | 38 |
| Streptophyta | N/A | Piperales | Piperaceae | 2 |
| Streptophyta | N/A | Proteales | Platanaceae | 1 |
| Streptophyta | Liliopsida | Poales | Poaceae | 330 |
| Streptophyta | N/A | Araucariales | Podocarpaceae | 5 |
| Streptophyta | N/A | Caryophyllales | Polygonaceae | 6 |
| Streptophyta | Polypodiopsida | Polypodiales | Polypodiaceae | 2 |
| Streptophyta | N/A | Caryophyllales | Portulacaceae | 1 |
| Streptophyta | Bryopsida | Pottiales | Pottiaceae | 1 |
| Chlorophyta | Palmophyllophyceae | Prasinococcales | Prasinococcaceae | 1 |
| Chlorophyta | Trebouxiophyceae | Prasiolales | Prasiolaceae | 1 |
| Streptophyta | N/A | Ericales | Primulaceae | 14 |
| Streptophyta | N/A | Proteales | Proteaceae | 1 |
| Chlorophyta | Ulvophyceae | Bryopsidales | Pseudocodiaceae | 1 |
| Chlorophyta | Chlorophyceae | Sphaeropleales | Pseudomuriellaceae | 1 |
| Streptophyta | Polypodiopsida | Psilotales | Psilotaceae | 1 |
| Streptophyta | Polypodiopsida | Polypodiales | Pteridaceae | 10 |
| Streptophyta | Jungermanniopsida | Ptilidiales | Ptilidiaceae | 1 |
| Chlorophyta | N/A | N/A | Pycnococcaceae | 1 |
| Streptophyta | N/A | Ranunculales | Ranunculaceae | 43 |
| Streptophyta | Polypodiopsida | Polypodiales | Rhachidosoraceae | 1 |
| Streptophyta | N/A | Rosales | Rhamnaceae | 4 |
| Streptophyta | N/A | Rosales | Rosaceae | 45 |
| Streptophyta | N/A | Gentianales | Rubiaceae | 6 |
| Streptophyta | N/A | Sapindales | Rutaceae | 15 |
| Streptophyta | N/A | Proteales | Sabiaceae | 2 |
| Streptophyta | N/A | Malpighiales | Salicaceae | 42 |
| Streptophyta | N/A | Santalales | Santalaceae | 1 |
| Streptophyta | N/A | Sapindales | Sapindaceae | 17 |
| Streptophyta | N/A | Ericales | Sapotaceae | 1 |
| Streptophyta | N/A | Saxifragales | Saxifragaceae | 5 |
| Chlorophyta | Chlorophyceae | Sphaeropleales | Scenedesmaceae | 2 |
| Streptophyta | N/A | Austrobaileyales | Schisandraceae | 5 |
| Streptophyta | Polypodiopsida | Schizaeales | Schizaeaceae | 2 |
| Chlorophyta | Chlorophyceae | Chaetophorales | Schizomeridaceae | 1 |
| Streptophyta | N/A | Santalales | Schoepfiaceae | 1 |
| Streptophyta | N/A | Cupressales | Sciadopityaceae | 1 |
| Streptophyta | N/A | Lamiales | Scrophulariaceae | 3 |
| Streptophyta | Lycopodiopsida | Selaginellales | Selaginellaceae | 1 |
| Chlorophyta | Chlorophyceae | Sphaeropleales | Selenastraceae | 1 |
| Streptophyta | N/A | Sapindales | Simaroubaceae | 2 |
| Streptophyta | N/A | Solanales | Solanaceae | 80 |
| Chlorophyta | Chlorophyceae | Sphaeropleales | Sphaeropleaceae | 1 |
| Streptophyta | Sphagnopsida | Sphagnales | Sphagnaceae | 1 |
| Streptophyta | Liliopsida | Pandanales | Stemonaceae | 5 |
| Streptophyta | N/A | Ericales | Styracaceae | 3 |
| Streptophyta | N/A | Ericales | Symplocaceae | 1 |
| Streptophyta | Liliopsida | Dioscoreales | Taccaceae | 1 |
| Streptophyta | Takakiopsida | Takakiales | Takakiaceae | 1 |
| Streptophyta | N/A | Caryophyllales | Talinaceae | 1 |
| Streptophyta | N/A | Huerteales | Tapisciaceae | 1 |
| Streptophyta | N/A | Cupressales | Taxaceae | 10 |
| Streptophyta | Tetraphidopsida | Tetraphidales | Tetraphidaceae | 1 |
| Streptophyta | N/A | Ericales | Theaceae | 20 |
| Streptophyta | Polypodiopsida | Polypodiales | Thelypteridaceae | 5 |
| Streptophyta | N/A | Malvales | Thymelaeaceae | 4 |
| Streptophyta | N/A | Apiales | Torricelliaceae | 1 |
| Chlorophyta | Trebouxiophyceae | Trebouxiales | Trebouxiaceae | 3 |
| Chlorophyta | Chlorophyceae | N/A | Treubariaceae | 1 |
| Bacillariophyta | Mediophyceae | Triceratiales | Triceratiaceae | 1 |
| Streptophyta | Liliopsida | Pandanales | Triuridaceae | 1 |
| Streptophyta | N/A | Trochodendrales | Trochodendraceae | 2 |
| Streptophyta | Liliopsida | Poales | Typhaceae | 1 |
| Chlorophyta | Ulvophyceae | Bryopsidales | Udoteaceae | 9 |
| Streptophyta | N/A | Rosales | Ulmaceae | 7 |
| Chlorophyta | Ulvophyceae | Ulvales | Ulvaceae | 4 |
| Streptophyta | N/A | Rosales | Urticaceae | 3 |
| Streptophyta | N/A | Santalales | Viscaceae | 4 |
| Streptophyta | N/A | Vitales | Vitaceae | 43 |
| Chlorophyta | Chlorophyceae | Chlamydomonadales | Volvocaceae | 1 |
| Streptophyta | N/A | Welwitschiales | Welwitschiaceae | 1 |
| Streptophyta | N/A | Canellales | Winteraceae | 1 |
| Streptophyta | Polypodiopsida | Polypodiales | Woodsiaceae | 2 |
| Streptophyta | N/A | Santalales | Ximeniaceae | 1 |
| Streptophyta | N/A | Cycadales | Zamiaceae | 7 |
| Streptophyta | Liliopsida | Zingiberales | Zingiberaceae | 4 |
| Streptophyta | Liliopsida | Alismatales | Zosteraceae | 1 |
| Streptophyta | Zygnemophyceae | Zygnematales | Zygnemataceae | 4 |
| Streptophyta | N/A | Zygophyllales | Zygophyllaceae | 2 |

###### Table S4 Comparison of tRNA genes predicted using tRNAscan-SE, ARAGORN against those in GenBank annotation (NC_000932.1). Please note there is no anticodon in the gene names of GenBank annotation.

There are 37 tRNA genes in the GenBank annotation, eight of them have introns. For these intron-containing tRNA genes, tRNAscan-SE failed to predict four genes completely and predicted the other four genes incorrectly. In contrast, ARAGORN predicted ten intron-containing tRNA genes, two of them are false positive, three of them differ with more than five bp from the reference. There are 29 tRNA genes in the GenBank annotations that donot have introns. The tRNAscan-SE predicted 29 tRNA genes without intron, 28 of them having no differences from their corresponding matches in the reference at the start and end postions. In contrast, ARAGORN predicted 29 tRNA genes, 22 of them having one to three bases difference from their matches in the reference at the start and end postions. It appears that tRNAscan-SE predicts better than ARAGORN for those genes without intron. In terms of gene names, tRNAscan-SE and ARAGORN predicted the two and five tRNA genes with wrong names, respectively. Both tools have difficulty in predicting the *trnG* gene for the position 8646-8679//9390-9432. It should be pointed out that there is one tRNA gene *trnS* (35312:35939) that is 628 bp long. Both tRNAscan-SE and ARAGORN predicted the tRNA gene to be *trnS*-UGA (35312:35403). It is very likely the GenBank annotation for the end position of this *trnS* gene is incorrect.

| **Results of tRNAscan-SE** | | | **Results of ARAGORN** | | | **GenBank annotation** | |
| --- | --- | --- | --- | --- | --- | --- | --- |
| **Gene** | **Location** | **Comparing results to those of GenBank** | **Gene** | **Location** | **Comparing results to those of GenBank** | **Gene** | **Location** |
| trnH-GUG | [4:76] (-) | + | trnH-GUG | [3:76] (-) | -1 | trnH | [4:76] (-) |
| Not applicable | | UP | trnK-UUU | [1716:1751//4311:4347] (-) | -1 | trnK | [1717:1751//4311:4347] (-) |
| trnQ-UUG | [6616:6687] (-) | + | trnQ-UUG | [6616:6687] (-) | + | trnQ | [6616:6687] (-) |
| trnS-GCU | [7785:7872] (-) | + | trnS-GCU | [7784:7872] (-) | -1 | trnS | [7785:7872] (+) |
| Not applicable | | UP | trnT-CGU | [8646:8679//9390:9432] (+) | WN, -19 | trnG | [8646:8668//9383:9431] (+) |
| trnR-UCU | [9590:9661] (+) | + | trnR-UCU | [9590:9661] (+) | + | trnR | [9590:9661] (+) |
| trnC-GCA | [27373:27443] (+) | + | trnC-GCA | [27373:27444] (+) | -1 | trnC | [27373:27443] (+) |
| trnD-GUC | [29801:29874] (-) | + | trnD-GUC | [29799:29875] (-) | -3 | trnD | [29801:29874] (-) |
| trnY-GUA | [30323:30406] (-) | + | trnY-GUA | [30322:30407] (-) | -2 | trnY | [30323:30406] (-) |
| trnE-UUC | [30466:30538] (-) | + | trnE-UUC | [30465:30539] (-) | -2 | trnE | [30466:30538] (-) |
| trnT-GGU | [31369:31440] (+) | + | trnT-GGU | [31368:31441] (+) | -2 | trnT | [31369:31440] (+) |
| trnS-UGA | [35312:35403] (-) | -- | trnS-UGA | [35312:35403] (-) | -- | trnS | [35312:35939] (+) |
| trnG-GCC | [36490:36560] (+) | + | trnG-GCC | [36490:36561] (+) | -1 | trnG | [36490:36560] (+) |
| trnM-CAU | [36704:36777] (-) | + | trnM-CAU | [36703:36777] (-) | -1 | trnfM | [36704:36777] (-) |
| trnS-GGA | [44827:44913] (+) | + | trnS-GGA | [44826:44914] (+) | -2 | trnS | [44827:44913] (+) |
| trnT-UGU | [46213:46285] (-) | + | trnT-UGU | [46212:46286] (-) | -2 | trnT | [46213:46285] (-) |
| Not applicable | | UP | trnL-UAA | [46894:46928//47441:47490] (+) | + | trnL | [46894:46928//47441:47490] (+) |
| trnF-GAA | [48175:48247] (+) | + | trnF-GAA | [48175:48247] (+) | + | trnF | [48175:48247] (+) |
| Not applicable | | UP | trnV-UAC | [51199:51233//51833:51871] (-) | + | trnV | [51199:51233//51833:51871] (-) |
| trnM-CAU | [52056:52128] (+) | + | trnM-CAU | [52056:52128] (+) | + | trnM | [52056:52128] (+) |
| trnW-CCA | [66229:66302] (-) | + | trnW-CCA | [66229:66302] (-) | + | trnW | [66229:66302] (-) |
| trnP-UGG | [66490:66563] (-) | + | trnP-UGG | [66488:66563] (-) | -2 | trnP | [66490:66563] (-) |
| trnM-CAU | [86312:86385] (-) | WN | trnM-CAU | [86311:86385] (-) | WN, -1 | trnI | [86312:86385] (-) |
| trnL-CAA | [94276:94356] (-) | + | trnL-CAA | [94275:94357] (-) | -2 | trnL | [94276:94356] (-) |
| trnV-GAC | [100709:100780] (+) | + | trnV-GAC | [100709:100780] (+) | + | trnV | [100709:100780] (+) |
| trnI-GAU | [102801:102836//102853:102888] (+) | -- | trnE-UUC | [102801:102832//103562:103601] (+) | WN, -10 | trnI | [102801:102837//103567:103601] (+) |
| trnA-UGC | [103665:103701//103742:103769] (+) | -- | trnA-UGC | [103665:103701//104503:104538] (+) | -1 | trnA | [103665:103702//104504:104538] (+) |
| Not applicable | | Not applicable | trnR-UCG | [105979:106038//108601:108635] (-) | OP | Not applicable | |
| trnR-ACG | [108302:108375] (+) | + | trnR-ACG | [108302:108376] (+) | -1 | trnR | [108302:108375] (+) |
| trnN-GUU | [109013:109084] (-) | + | trnN-GUU | [109012:109085] (-) | -1 | trnN | [109013:109084] (-) |
| trnL-UAG | [114270:114349] (+) | + | trnL-UAG | [114269:114350] (+) | -2 | trnL | [114270:114349] (+) |
| trnN-GUU | [129565:129636] (+) | + | trnN-GUU | [129564:129637] (+) | -2 | trnN | [129565:129636] (+) |
| Not applicable | | Not applicable | trnR-UCG | [130014:130048//132611:132670] (+) | OP | Not applicable | |
| trnR-ACG | [130274:130347] (-) | + | trnR-ACG | [130273:130347] (-) | -1 | trnR | [130274:130347] (-) |
| trnA-UGC | [134880:134946//134909:134984] (-) | -- | trnA-UGC | [134111:134146//134948:134984] (-) | -2 | trnA | [134111:134145//134947:134984] (-) |
| trnI-GAU | [135761:135811//135798:135848] (-) | -- | trnE-UUC | [135048:135087//135817:135848] (-) | WN, -10 | trnI | [135048:135082//135812:135848] (-) |
| trnV-GAC | [137869:137940] (-) | + | trnV-GAC | [137869:137940] (-) | + | trnV | [137869:137940] (-) |
| trnL-CAA | [144293:144373] (+) | + | trnL-CAA | [144292:144374] (+) | -2 | trnL | [144293:144373] (+) |
| trnM-CAU | [152264:152337] (+) | WN | trnM-CAU | [152264:152338] (+) | WN, -1 | trnI | [152264:152337] (+) |

+: the prediction is identical to that in GenBank annotation;

--: the difference at the start and end position is more than five bases;

-number: the two prediction results differ at the start and end positions in the numbers of bases;

UP: under prediction;

OP: over prediction;

WN: wrong name.

###### Table S5 Summary of the annotation results of five different web servers for the plastome sequence of *A. thaliana*

| **Type of Errors** | **GenBank annotation** | **Annotation Results from Different Web Servers** | | | | |
| --- | --- | --- | --- | --- | --- | --- |
|  |  | **AGORA** | **GeSeq** | **Mfannot** | **DOGMA** | **CPGAVAS2** |
| No. of incorrectly named genes | 0 | 0 | 1 (*pbf1*) | 3 (*orf526, orf160, ftsH*) | 0 | 0 |
| No. of protein-coding genes missing | 0 | 0 | 0 | 2 (*rpl23*, one copy of *rps12*) | 1 (one copy of *ycf1*) | 0 |
| Errors in the start and end of protein coding genes | 0 | 11 (*ycf3, clpP, petB, petD, rpl16, rpl2, ndhB, ndhA, rps12, psbJ, rps11*) | 5 (*petB, petD, psbC, rpl16, ycf1*) | 11 (*accD, matK, ndhB, ndhD, petB, petD, psbK, rpl16, rps12, rps16, ycf1*) | 13 (*matK, ndhB, ndhD, ndhK, petB, petD, petN, psbC, psbI, rpl16, rps12, rps16, ycf1*) | 2 (*ycf1, ndhD*) |
| No. of genes missing exons | 0 | 9 (*ycf3, clpP, petB, petD, rpl16, rpl2, ndhB, ndhA, rps12*) | 3 (*petB, petD, rpl16*) | 5 (*petB, petD, rpl16, rps12, rps16*) | 6 (*ndhA, petB, petD, rpl16, rps12, rps16*) | 0 |
| No. of *petB* exon | 2 | 1 | 1 | 1 | 1 | 2 |
| No. of *petD* exon | 2 | 1 | 1 | 1 | 1 | 2 |
| No. of *rpl16* exon | 2 | 1 | 1 | 1 | 1 | 2 |
| No. of *rps12* exon | 3 (joined) | 1 | 3 (not joined ) | 2 (joined) | 3 (not joined) | 3 (joined) |
| No. of rRNA gene | 7 (missing one copy of *rrn5S*) | 8 | 8 | 6 (missing *rrn4.5S*) | 8 | 8 |

###### Table S6 Summary of the annotation results of five different web servers for the plastome sequence of *G. longituha*

| **Type of Errors** | **Annotation after RNA-seq correction** | **Annotation Results from Different Web Servers** | | | | |
| --- | --- | --- | --- | --- | --- | --- |
|  |  | **AGORA** | **GeSeq** | **MFannot** | **DOGMA** | **CPGAVAS2** |
| No. of incorrectly named genes | 0 | 0 | 0 | 7(*ycf68, orf42, orf55, orf188, orf56, orf42,psbG*) | 6 (*orf510, orf155, ftsH, rns, rnl, orf186*) | 0 |
| No. of protein-coding genes missing | 0 | 0 | 1 (*ycf15*) | 0 | 5 (*rpl23, ycf2, ycf15, rps12, ycf1*) | 0 |
| No. of genes missing exons | 0 | 8 (*ycf3, clpP, petB, petD, rpl16, rpl2, ndhB, rps12*) | 3 (*rpl16, petB, petD*) | 5 (*rps16, rpl16,petB, petD, rps12*) | 5 (*rps16, petB, petD, rpl16, ndhB*) | 0 |
| Errors in the start and end of protein coding genes | 0 | 21 (*matK, rpoC1, petN, ycf3, atpE, rbcL, accD, psbJ, rps12, clpP, psbH, petB, petD, rpl16, rpl2, rpl23, ycf2, ndhB, ycf1, ndhD, ndhA*) | 18 (*psbA, matK, petN, ndhK, rbcL, accD, psbH, ycf1, psbH, rps3, rpl22, rpl23, rps12, ndhF, rpl32, ccsA, ndhD, ndhI*) | 20 (*psbA, matK, psbI, rpoC1, psbC, atpE, rbcL, psbB, psbT, psbH, rps3, rpl32, ccsA, ndhD, ndhE, ndhI, ndhA, ycf1, ycf15, rpl23*) | 5 (*psbK, ndhK, accD, rps12, psbH*) | 5 (*petN, psbH, petB, ndhF, ycf1)* |
| No. of *petB* exon | 2 | 1 | 1 | 1 | 1 | 2 |
| No. of *petD* exon | 2 | 1 | 1 | 1 | 1 | 2 |
| No. of *rpl16* exon | 2 | 1 | 1 | 1 | 1 | 2 |
| No. of *rps12* exon | 3 (joined) | 1 | 3 (not joined) | 2 (joined) | 1 | 3 (joined) |
| No. of rRNA genes | 8 | 8 | 8 | 4 | 8 | 8 |

###### Figure S1 Differences in the translation start sites for *matK* genes from thirteen plastomes of *Arabidopsis* genus

The sequences of *matK* gene for 13 *Arabidopsis* species *A. lyrata* subsp. *Lyrata, A. pedemontana, A. arenicola, A. umezawana, A. arenosa, A. halleri, A. thaliana, A. petrogena, A. cebennensis, A. neglecta, A. lyrata, A. croatica*, and *A. suecica* were aligned using CodonCode Aligner (upper panel) and MEGA X (lower panel). The nucleotides are shaded in different color. ‘A’: green; ‘G’: pink; ‘C’: blue; ‘T’: red.


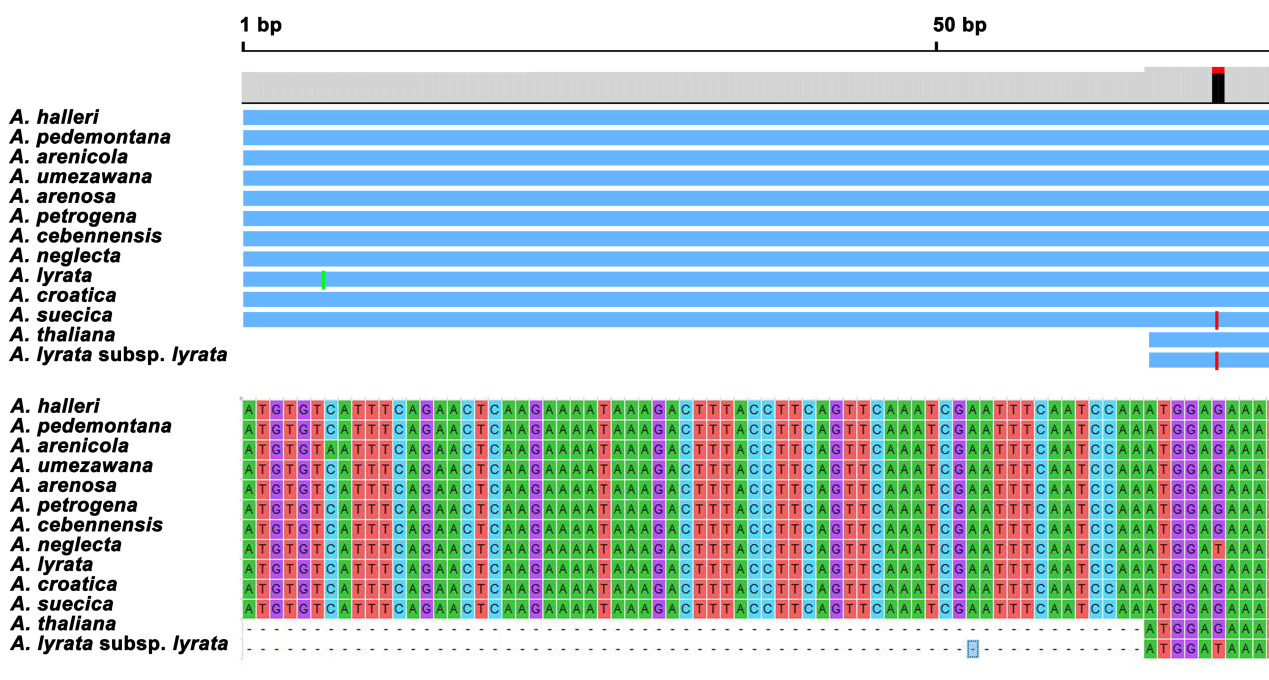


###### Figure S2 Missing one 5S rRNA gene in the GenBank annotation for the plastome of *A. thaliana*

Panel A shows the seven rRNA genes described in NCBI RefSeq NC_000932.1, Panel B shows the alignment of sequences from positions 130700-130580 coded on the negative strand to 107949-108069 coded on the positive strand. The sequences of these two fragments are identical.


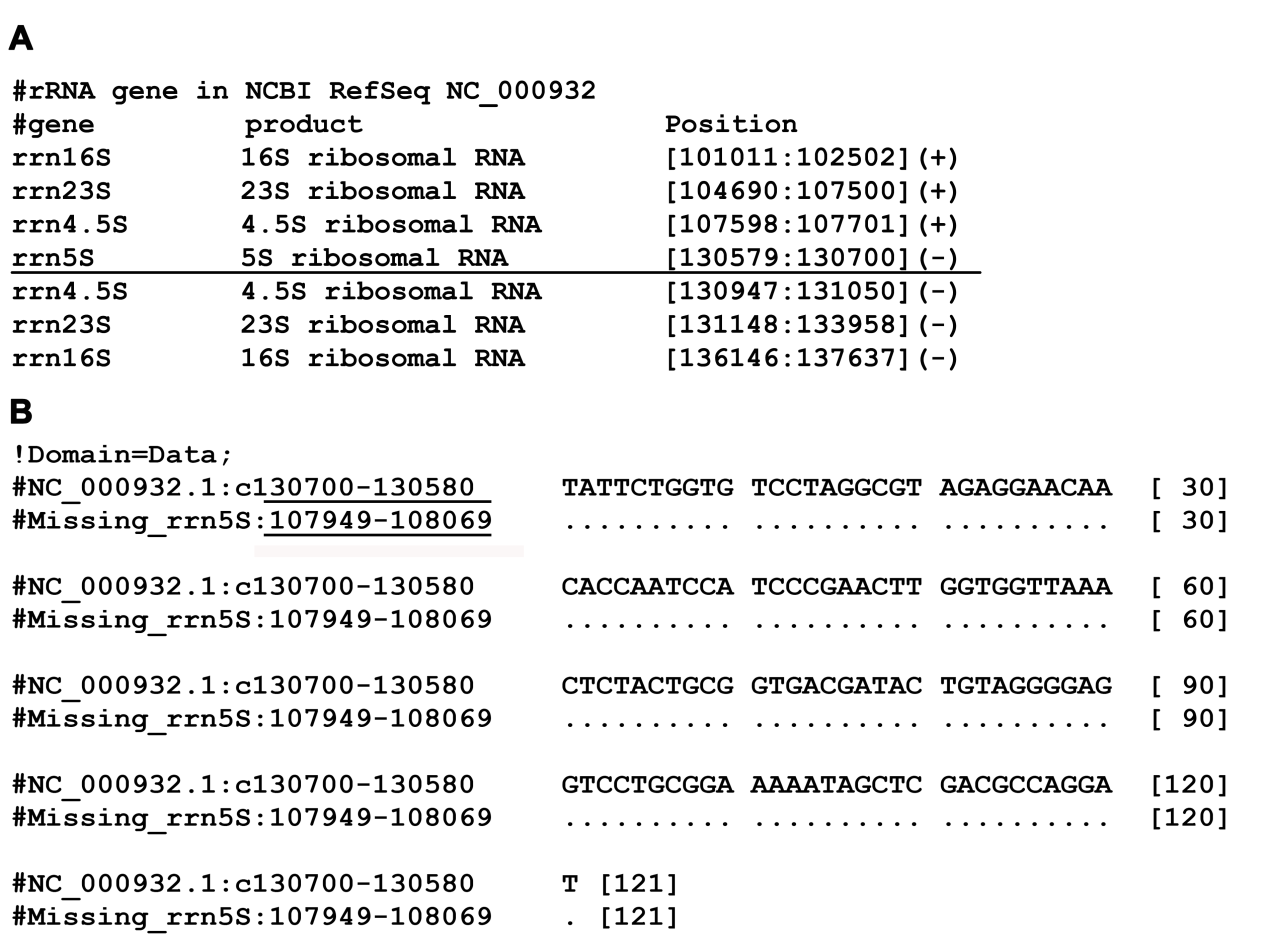


###### Figure S3 Graphic representation of RNA-seq reads mapped to exon-intron boundaries of (A) 3’ of *ndhA* intron, (B) 5’ of *ndhA* intron and (C) 3’ of *petD* intron from *Medicago truncatula*. The *ndhA* and *petD* are also encoded on the negative strand as shown in Figure 2.


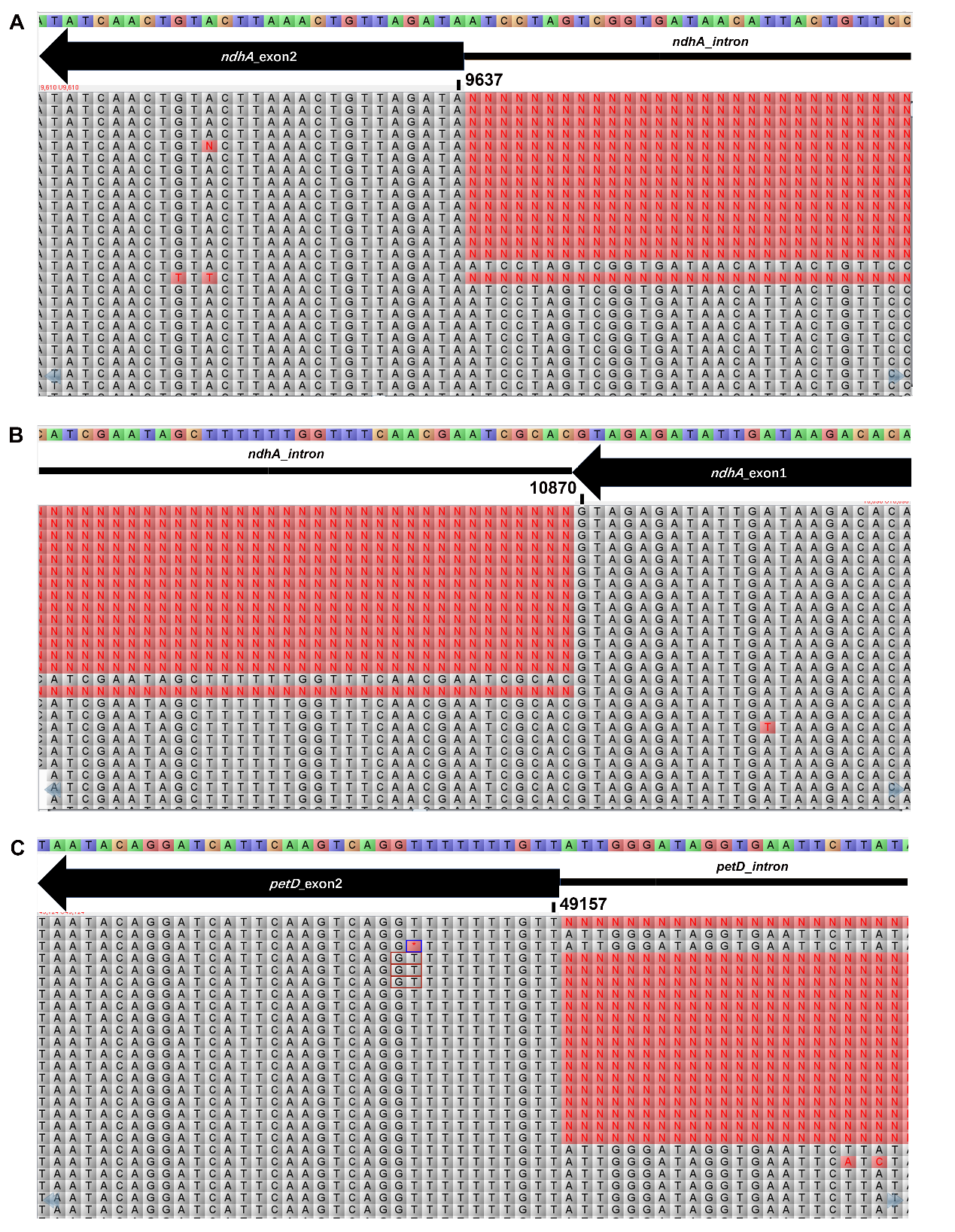


###### Figure S4 Alignment and statistics of the sequences of the small exons found in (A) *petB*; (B) *petD* and (C) *rpl16* from 2544 plastome sequences.

The patterns and their corresponding counts are shown. Those pattern having frequency less than 2 are not counted.


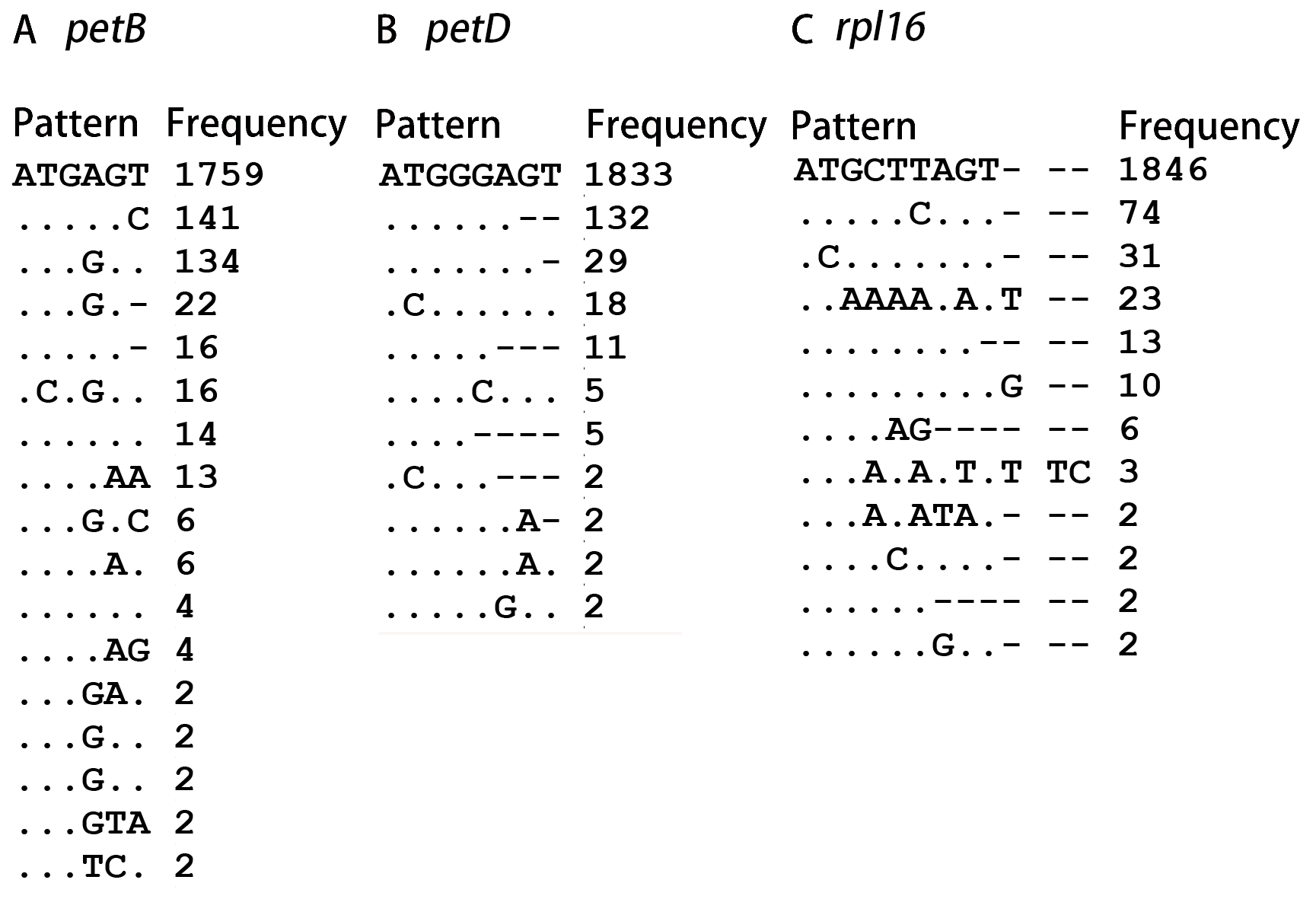


###### Figure S5 Graphic representation of RNA-seq reads mapped to the small exons and 5’ UTR of (A) *petB*, (B) *petD* and (C) *rpl16* genes from *A. thaliana*. Arrows indicates the direction from 5’ to 3’. The numbers indicate the start and end positions of the exons.


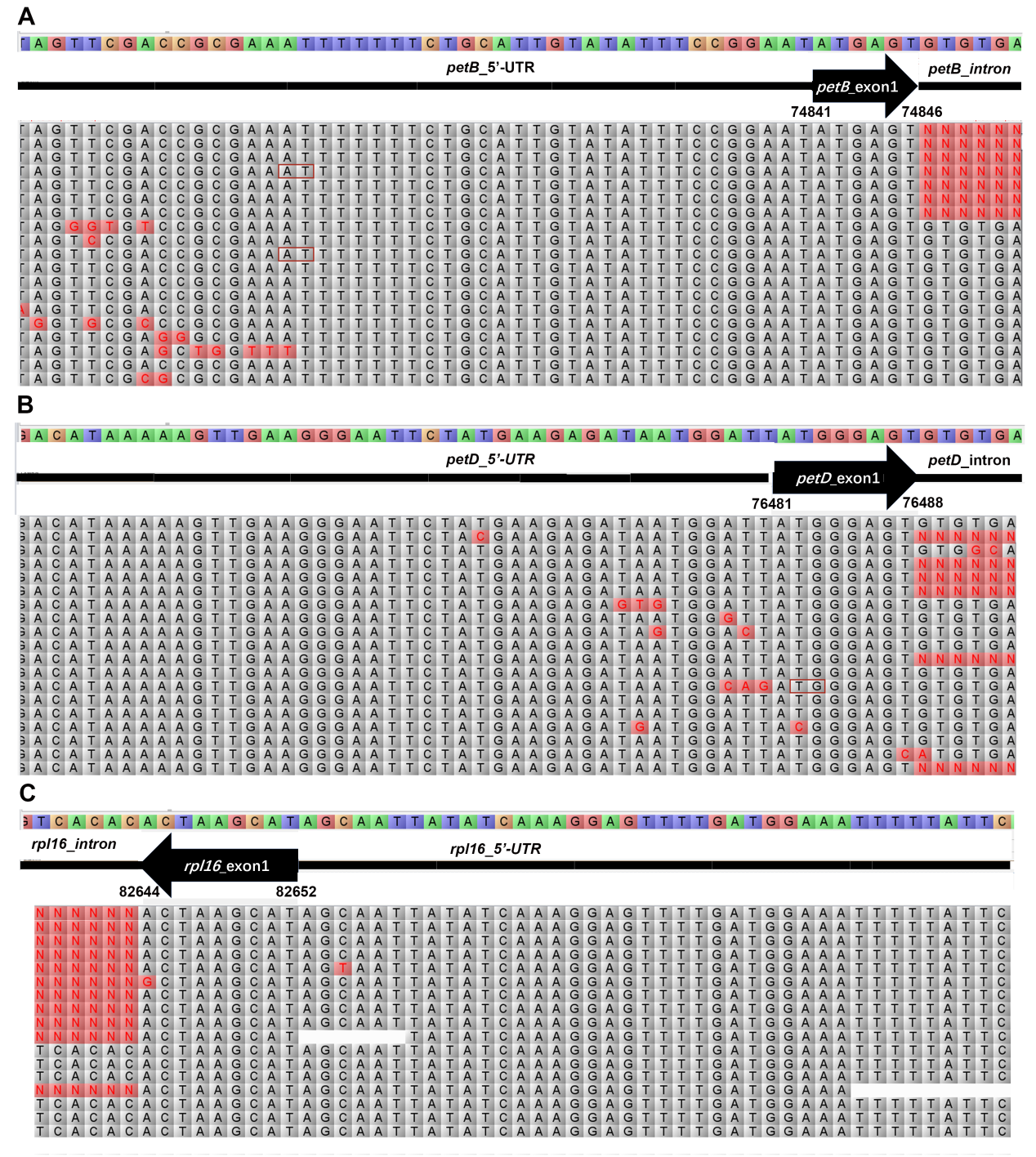


###### Figure S6 Alignment of the 50 bp 5’ UTR sequence upstream of start codon, and the first six to nine bases in the CDS sequences of the gene (A) *petB*, (B) *petD* and (C) *rpl16* from 79, 71 and 80 Brassicaceae plants.

The Latin names and accession numbers from GenBank are shown on the left. The corresponding sequences are shown on the right. The sequence on the top is the reference sequence. The CDS for the small exons are in bold and underlined. “.”: identical; “-”: indel.

A *(petB)*

Aethionema_arabicum_NC_034367 TTTGGTAGTT CGACCGCA-A AATTTTTTGC TTTCTGCATT GTATATTTCC GGAAT**ATGAG T**

Aethionema_cordifolium_NC_009265 .......... ........-. ........T. .......... .......... .......... .

Aethionema_grandiflorum_NC_009266 .......... .......G-. ........T. .......... .......... .......... .

Alyssum_desertorum_NC_034299 .......... .......G-- ..A....--- .......... .......... .......... .

Arabidopsis_arenicola_NC_030346 .......... ..G....G-- ..AA...--- .......... .......... .......... .

Arabidopsis_arenosa_NC_029334 .......... ..G....G-- ..AA...--- .......... .......... .......... .

Arabidopsis_cebennensis_NC_029335 .......... ..G....G-- ..AA...--- .......... .......... .......... .

Arabidopsis_croatica_NC_030347 .......... ..G....G-- ..AA...--- .......... .......... .......... .

Arabidopsis_halleri_NC_034366 .......... ..G....G-- ..AA...--- .......... .......... .......... .

Arabidopsis_lyrata_NC_034365 .......... ..G....G-- ..AA...--- .......... .......... .......... .

Arabidopsis_lyrata_subsp._lyrata_NC_034379 .......... ..G....G-- ..AA...--- .......... .......... .......... .

Arabidopsis_neglecta_NC_030348 .......... ..G....G-- ..AA...--- .......... .......... .......... .

Arabidopsis_pedemontana_NC_029336 .......... ..G....G-- ..AA...--- .......... .......... .......... .

Arabidopsis_petrogena_NC_030349 .......... ..G....G-- ..AA...--- .......... .......... .......... .

Arabidopsis_suecica_NC_030350 .......... .......G-- ..A....--- .......... .......... .......... .

Arabidopsis_thaliana_NC_000932 .......... .......G-- ..A....--- .......... .......... .......... .

Arabidopsis_umezawana_NC_030351 .......... ..G....G-- ..AA...--- .......... .......... .......... .

Arabis_flagellosa_NC_037475 .......... .......G-- ..A...---- .......... .......... .......... .

Arabis_hirsuta_NC_009268 .......... .......G-- ..A...---- .......... .......... .......... .

Barbarea_verna_NC_009269 .......... .......G-- ..A.....-- .......... .......... .......... .

Biscutella_baetica_NC_039952 .......... .......GA. ..A....--- .......... .......... .......... .

Biscutella_lyrata_NC_039953 .......... .......G-. ..A....--- .G........ .......... .......... .

Brassica_napus_NC_016734 .......... .......G-- ..A....--- .......... .......... .......... .

Brassica_nigra_NC_030450 .......... .......G-- ..A....--- .......... .......... .......... .

Braya_humilis_NC_035515 .......... .......G-- ..A....--- .......... .......... .......... .

Bunias_erucago_NC_036110 .......... .......G-- ..A....--- .......... .......... .......... .

Bunias_orientalis_NC_036111 .......... .......G-- G.A....--- .......... .......... .......... .

Camelina_sativa_NC_029337 .......... .......G-- ..A..A.--- .......... .......... .......... .

Capsella_bursa-pastoris_NC_009270 .......... ........-- ..A....--- .......... .......... .......... .

Capsella_grandiflora_NC_028517 .......... ........-- ..A....--- .......... .......... .......... .

Capsella_rubella_NC_027693 .......... ........-- ..A....--- .......... .......... .......... .

Cochlearia_borzaeana_NC_029253 .......... .......G-- ..A....--- .......... .......... .......... .

Cochlearia_islandica_NC_029254 .......... .......G-- ..A....--- .......... .......... .......... .

Cochlearia_pyrenaica_NC_029331 .......... .......G-- ..A....--- .......... .......... .......... .

Cochlearia_tridactylites_NC_029332 .......... .......G-- ..A....--- .......... .......... .......... .

Crucihimalaya_wallichii_NC_009271 .......... .......G-- ..A.....-- .......... .......... .......... .

Draba_nemorosa_NC_009272 .......... .......G-- ..A...---- .......... .......... .......... .

Draba_oreades_NC_037760 .......... .......G-- ..A...---- .......... .......... .......... .

Eutrema_botschantzevii_NC_029379 .......... .......G-- ..A....--- .......... .......... .......... .

Eutrema_halophilum_NC_029378 .......... .......G-- ..A....--- .......... .......... .......... .

Eutrema_heterophyllum_NC_028728 C......... .......G-- ..A....--- .......... .......... .......... .

Eutrema_salsugineum_NC_028170 .......... .......G-- ..A....--- .......... .......... .......... .

Eutrema_yunnanense_NC_028727 C......... ........-- ..A....--- .......... .......... .......... .

Heldreichia_bupleurifolia_NC_039954 .......... .......G-- ..A....--- .......... .......... .......... .

Hesperis_matronalis_NC_035511 .......... T......G-- ..A.....-- .......... .......... .......... .

Hesperis_sylvestris_NC_035512 .......... .......G-- ..A.....-- .......... .......... .......... .

Ionopsidium_acaule_NC_029333 .......... .......G-- ..A....--- .......... .......... .......... .

Isatis_tinctoria_NC_028415 .......... .......G-- ..A....--- .......... .......... .......... .

Lepidium_meyenii_NC_034363 .......... .......G-- ..A.....-- .......... .......... .......... .

Lepidium_virginicum_NC_009273 .......... .......G-- ..A....--- .......... .......... .......... .

Lobularia_libyca_NC_035513 .......... .......G-- ..A....--- .......... .......... .......... .

Lobularia_maritima_NC_009274 .......... .......G-- ..A....--- .......... .......... .......... .

Lunaria_rediviva_NC_039955 .......... .......G-- ........-- .......... .......... .......... .

Matthiola_incana_NC_034358 .......... .......G-- ..A.....-- .......... .......... .......... .

Megacarpaea_delavayi_NC_034360 .......... .......G-- ..A....--- .......... .......... .......... .

Megadenia_pygmaea_NC_034357 .......... .......G-- ..A.....-- .......... .......... .......... .

Morettia_canescens_NC_035514 .......... .......G-- ..A.....-- .......... .......... .......... .

Nasturtium_officinale_NC_009275 .......... .......G-- ..A....--- .......... .......... .......... .

Neotorularia_korolkowii_NC_034361 .......... .......G-- ..A.C..--- .......... .......... .......... .

Olimarabidopsis_pumila_NC_009267 .......... .......G-- ..A....--- .......... .......... .......... .

Orychophragmus_diffusus_NC_033498 .......... .......G-- ..A....--- .......... .......... .......... .

Orychophragmus_hupehensis_NC_033500 .......... .......G-- ..A....--- .......... .......... .......... .

Orychophragmus_taibaiensis_NC_033499 .......... .......G-- ..A....--- .......... .......... .......... .

Pachycladon_cheesemanii_NC_021102 .......... .......G-- ..A.....-- .......... .......... .......... .

Pachycladon_enysii_NC_018565 .......... .......G-. ..A....--- .......... .......... .......... .

Pugionium_cornutum_NC_030516 .......... .......G-- ..A....--- .......... .......... .......... .

Pugionium_dolabratum_NC_030515 .......... .......G-- ..A....--- .......... .......... .......... .

Raphanus_sativus_NC_024469 .......... T......G-- ..A.....-- .......... .......... .......... .

Ricotia_aucheri_NC_039956 G......... ....T..G-. ..AC...--- .......... .......... .......... .

Ricotia_carnosula_NC_039957 .......... .......G-. ..A....--- ...A...... .......... .......... .

Ricotia_cretica_NC_039958 .......... .......G-. ..A....--- .......... .......... .......... .

Ricotia_davisiana_NC_039959 .......... .......G-. ..A....--- .......... .......... .......... .

Ricotia_isatoides_NC_039960 ....A..... .......G-. ..A....--- .......... .......... .......... .

Ricotia_lunaria_NC_039961 .......... .C.....G-. ..A....--- .......... .......... .......... .

Schrenkiella_parvula_NC_028726 .......... .......G-- ..A....--- ...T...... .......... .......... .

Sinalliaria_limprichtiana_NC_034287 .......... .......G-- ..A....--- .......... .......... .......... .

Sinalliaria_limprichtiana_var._grandifolia_NC_034286 .......... .......G-- ..A....--- .......... .......... .......... .

Sinapis_arvensis_NC_035303 .......... .......G-- ..G....--- .......... .......... .......... .

Sisymbrium_irio_NC_037838 .......... .......G-- ..A....--- .......... .......... .......... .

Solms-laubachia_eurycarpa_NC_034359 .......... .......G-- ..A.C..--- ...A..A... -......... .......... .

Thlaspi_arvense_NC_034362 .......... .......G-- ..A....--- G......... .......... .......... .

B *(petD)*

Aethionema_arabicum_NC_034367 ATTTTTGGC AT-AAATAGT TGAAGGGAAT TCTATGAAGA GAAAATGGAT T**ATGGGAGT**

Aethionema_grandiflorum_NC_009266 ......... ..-...A... G......... .......... .......... .........

Aethionema_cordifolium_NC_009265 ......... ..-....... G......... .......... .......... .........

Lobularia_libyca_NC_035513 ......TA. .G-...A... .......... .......... ....G..... .........

Sisymbrium_irio_NC_037838 .......AA ..-...A... .......... .....A.... .......... .........

Solms-laubachia_eurycarpa_NC_034359 ......TA. .GA...A... .......... .......... .......... .........

Ricotia_lunaria_NC_039961 .......A. ..-...A... .......... .......... .......... .........

Ionopsidium_acaule_NC_029333 ......TA. ..-...A... G......... .......... .......... .........

Bunias_orientalis_NC_036111 ......TA. ..-...A... .......... .......... .......... .........

Draba_oreades_NC_037760 .......A. .G-...A... .......... .......... ......A... .........

Draba_nemorosa_NC_009272 .......A. .G-...A... .......... .......... .......... .........

Braya_humilis_NC_035515 ......TA. ..-...A... .......... .......... ..C....... .........

Ricotia_aucheri_NC_039956 ......TAT ..-...A... .......... .......... .......... .........

Bunias_erucago_NC_036110 ......TA. ..-...A... .......... .......... .......... .........

Cochlearia_borzaeana_NC_029253 ......TA. ..-...A... .......... .......... .......... .........

Cochlearia_islandica_NC_029254 ......TA. ..-...A... .......... .......... .......... .........

Cochlearia_pyrenaica_NC_029331 ......TA. ..-...A... .......... .......... .......... .........

Cochlearia_tridactylites_NC_029332 ......TA. ..-...A... .......... .......... .......... .........

Eutrema_yunnanense_NC_028727 ......TA. ..-...A... .......... .......... .......... .........

Hesperis_matronalis_NC_035511 ......TA. ..-...A... .......... .......... .......... .........

Hesperis_sylvestris_NC_035512 ......TA. ..-...A... .......... .......... .......... .........

Lobularia_maritima_NC_009274 ......TA. ..-...A... .......... .......... .......... .........

Matthiola_incana_NC_034358 ......TA. ..-...A... .......... .......... .......... .........

Morettia_canescens_NC_035514 ......TA. ..-...A... .......... .......... .......... .........

Pugionium_cornutum_NC_030516 ......TA. ..-...A... .......... .......... .......... .........

Pugionium_dolabratum_NC_030515 ......TA. ..-...A... .......... .......... .......... .........

Ricotia_isatoides_NC_039960 .......A. ..-...AG.. .......... .......... .......... .........

Olimarabidopsis_pumila_NC_009267 .......A. ..-...A... .......... .......... ..T....... .........

Camelina_sativa_NC_029337 .......A. ..-...AG.. .......... .......... ..T....... .........

Crucihimalaya_wallichii_NC_009271 .......A. ..-...A... ..G....... .......... ..T....... .........

Pachycladon_cheesemanii_NC_021102 .......AT ..-...A... .......... .......... ..T....... .........

Arabidopsis_arenicola_NC_030346 .......A. ..-...A... .......... .......... ..T....... .........

Arabidopsis_arenosa_NC_029334 .......A. ..-...A... .......... .......... ..T....... .........

Arabidopsis_cebennensis_NC_029335 .......A. ..-...A... .......... .......... ..T....... .........

Arabidopsis_croatica_NC_030347 .......A. ..-...A... .......... .......... ..T....... .........

Arabidopsis_lyrata_subsp._lyrata_NC_034379 .......A. ..-...A... .......... .......... ..T....... .........

Arabidopsis_neglecta_NC_030348 .......A. ..-...A... .......... .......... ..T....... .........

Arabidopsis_pedemontana_NC_029336 .......A. ..-...A... .......... .......... ..T....... .........

Arabidopsis_petrogena_NC_030349 .......A. ..-...A... .......... .......... ..T....... .........

Arabidopsis_suecica_NC_030350 .......A. ..-...A... .......... .......... ..T....... .........

Arabidopsis_thaliana_NC_000932 .......A. ..-...A... .......... .......... ..T....... .........

Arabidopsis_umezawana_NC_030351 .......A. ..-...A... .......... .......... ..T....... .........

Capsella_bursa-pastoris_NC_009270 .......A. ..-...A... .......... .......... ..T....... .........

Capsella_grandiflora_NC_028517 .......A. ..-...A... .......... .......... ..T....... .........

Capsella_rubella_NC_027693 .......A. ..-...A... .......... .......... ..T....... .........

Pachycladon_enysii_NC_018565 .......A. ..-...A... .......... .......... ..T....... .........

Megadenia_pygmaea_NC_034357 .......A. ..-...A... .......... .A........ .......... .........

Megacarpaea_delavayi_NC_034360 ......TTT T.-----... .......... .A........ .......... .........

Neotorularia_korolkowii_NC_034361 ......TA. ..A...A... .......... .A........ .......... .........

Barbarea_verna_NC_009269 G......A. ..-...A... .......... .......... .......... .........

Nasturtium_officinale_NC_009275 G......A. ..-...A... .......... .......... .......... .........

Arabis_flagellosa_NC_037475 .......A. ..-..GA... .......... .......... .......... .........

Arabis_hirsuta_NC_009268 .......A. ..-..GA... .......... .......... .......... .........

Lepidium_virginicum_NC_009273 .......C. ..-...A... .......... .......... .......... .........

Sinalliaria_limprichtiana_NC_034287 .......A. C.-...A... .......... .......... .......... .........

Biscutella_baetica_NC_039952 .......A. ..-...A... .......... .......... .......... .........

Biscutella_lyrata_NC_039953 .......A. ..-...A... .......... .......... .......... .........

Brassica_napus_NC_016734 .......A. ..-...A... .......... .......... .......... .........

Brassica_nigra_NC_030450 .......A. ..-...A... .......... .......... .......... .........

Heldreichia_bupleurifolia_NC_039954 .......A. ..-...A... .......... .......... .......... .........

Isatis_tinctoria_NC_028415 .......A. ..-...A... .......... .......... .......... .........

Lepidium_meyenii_NC_034363 .......A. ..-...A... .......... .......... .......... .........

Lunaria_rediviva_NC_039955 .......A. ..-...A... .......... .......... .......... .........

Orychophragmus_diffusus_NC_033498 .......A. ..-...A... .......... .......... .......... .........

Orychophragmus_hupehensis_NC_033500 .......A. ..-...A... .......... .......... .......... .........

Orychophragmus_taibaiensis_NC_033499 .......A. ..-...A... .......... .......... .......... .........

Raphanus_sativus_NC_024469 .......A. ..-...A... .......... .......... .......... .........

Ricotia_carnosula_NC_039957 .......A. ..-...A... .......... .......... .......... .........

Ricotia_cretica_NC_039958 .......A. ..-...A... .......... .......... .......... .........

Ricotia_davisiana_NC_039959 .......A. ..-...A... .......... .......... .......... .........

Sinapis_arvensis_NC_035303_7_1 .......A. ..-...A... .......... .......... .......... ........-

C *(rpl16)*

Aethionema_arabicum_NC_034367 AATAAGGTT GAATAAAAAT TTCCATCAAA ACTCCTTTGA TATAATTGCT **ATGCTTAGT**

Aethionema_cordifolium_NC_009265 ......... .......... .......... .......... .......... .........

Aethionema_grandiflorum_NC_009266 ......A.. .......... .......... .......... .......... .........

Arabidopsis_arenicola_NC_030346 ......A.. .......... .......... ...A...... .......... .........

Arabidopsis_arenosa_NC_029334 ......A.. .......... .......... ...A...... .......... .........

Arabidopsis_cebennensis_NC_029335 ......A.. .......... .......... ...A...... .......... .........

Arabidopsis_croatica_NC_030347 ......A.. .......... .......... ...A...... .......... .........

Arabidopsis_halleri_NC_034366 ......A.. .......... .......... ...A...... .......... .........

Arabidopsis_lyrata_NC_034365 ......A.. .......... .......... ...A...... .......... .........

Arabidopsis_lyrata_subsp._lyrata_NC_034379 ......A.. .......... .......... ...A...... .......... .........

Arabidopsis_neglecta_NC_030348 ......A.. .......... .......... ...A...... .......... .........

Arabidopsis_pedemontana_NC_029336 ......A.. .......... .......... ...A...... .......... .........

Arabidopsis_petrogena_NC_030349 ......A.. .......... .......... ...A...... .......... .........

Arabidopsis_suecica_NC_030350 ......A.. .......... .......... .......... .......... .........

Arabidopsis_thaliana_NC_000932 ......A.. .......... .......... .......... .......... .........

Arabidopsis_umezawana_NC_030351 ......A.. .......... .......... ...A...... .......... .........

Arabis_flagellosa_NC_037475 ......... .......... ..T....... .......... .......... .........

Arabis_hirsuta_NC_009268 ......... .......... ..T....... .......... .......... .........

Barbarea_verna_NC_009269 ......... .......... .......... .......... .......... .........

Biscutella_baetica_NC_039952 ......A.. .......... .......... .......... .......... .........

Biscutella_lyrata_NC_039953 ......A.. .......... .......... .......... .......... .........

Brassica_napus_NC_016734 ......... .......C.. .......... .......... .......... .........

Brassica_nigra_NC_030450 ......... .......C.. ......G... .......... .......... .........

Braya_humilis_NC_035515 ......... .......... .......... .......... .......... .........

Bunias_erucago_NC_036110 ......... .......... .......... .......... .......... .........

Bunias_orientalis_NC_036111 ......... .......... .......... .........G .......... .........

Cakile_arabica_NC_030775 ......... .......... .......... .......... .......... .........

Camelina_sativa_NC_029337 ......A.. .......... .......... .......... .......... .........

Capsella_bursa-pastoris_NC_009270 ......A.. T......... .......... .......... .......... .........

Capsella_grandiflora_NC_028517 ......A.. T......... .......... .......... .......... .........

Capsella_rubella_NC_027693 ......A.. T......... .......... .......... .......... .........

Cochlearia_borzaeana_NC_029253 ......A.. T......... .......... .......... .......... .........

Cochlearia_islandica_NC_029254 ......A.. T......... .......... .......... .......... .........

Cochlearia_pyrenaica_NC_029331 ......A.. T......... .......... .......... .......... .........

Cochlearia_tridactylites_NC_029332 ......A.. T......... .......... .......... .......... .........

Crucihimalaya_wallichii_NC_009271 ......... .......... .......... .......... .......... .........

Draba_nemorosa_NC_009272 ......... .......... ..T....... .......... .....G.... .........

Draba_oreades_NC_037760 ......... .......... ..T....... .......... .......... .........

Eutrema_botschantzevii_NC_029379 ......... T......... .......... .......... .......... .........

Eutrema_halophilum_NC_029378 ......... T......... .......... .......... .......... .........

Eutrema_heterophyllum_NC_028728 ......... T......... .......... .......... .......... .........

Eutrema_salsugineum_NC_028170 ......... T......... ....C..... .......... .......... .........

Eutrema_yunnanense_NC_028727 ......... T......... .......... .......... .......... .........

Heldreichia_bupleurifolia_NC_039954 ......A.. .......... .......... .......... .......... .........

Hesperis_matronalis_NC_035511 ......... .......... .......... ....------ --........ .........

Hesperis_sylvestris_NC_035512 ......... .......... .......... ....------ --........ .........

Ionopsidium_acaule_NC_029333 ......A.. T......... .......... .......... .......... .........

Isatis_tinctoria_NC_028415 ......... .......... .......... .......... .......... .........

Lepidium_meyenii_NC_034363 ......... .......... .......... .......... .......... .........

Lepidium_virginicum_NC_009273 ......... .......... .......... .......... .......... .........

Lobularia_libyca_NC_035513 ......... .......... .......... ...T...... .......... .........

Lobularia_maritima_NC_009274 ......... .......... .......... ...T...... .......... .........

Lunaria_rediviva_NC_039955 ......A.. .......... ...T...... ..C....... .......... .........

Matthiola_incana_NC_034358 ......... .......... .......... .......... .......... .........

Megacarpaea_delavayi_NC_034360 ......... ......T... .......... ....T..... .......... .........

Megadenia_pygmaea_NC_034357 ......... .......... .......... .......... .......... .........

Morettia_canescens_NC_035514 ......... .......... .......... ...T...... .......... .........

Nasturtium_officinale_NC_009275 ......... .......... .......... .......... .......... .........

Neotorularia_korolkowii_NC_034361 .....A... A......... .......T.. .......... .......... .........

Olimarabidopsis_pumila_NC_009267 ......... A......... ......A... .......... .......... .........

Orychophragmus_diffusus_NC_033498 ......... .......... .......... .......... .......... .........

Orychophragmus_hupehensis_NC_033500 ......... .......... .......... .......... .......... .........

Orychophragmus_taibaiensis_NC_033499 ......... .......... .......... .......... .......... .........

Pachycladon_cheesemanii_NC_021102 ......... .......... .......... .......... .......... .........

Pachycladon_enysii_NC_018565 ......... .......... .......... .......... .......... .........

Pugionium_cornutum_NC_030516 ......... .......... .......... .......... .......... .........

Pugionium_dolabratum_NC_030515 ......... .......... .......... .......... .......... .........

Raphanus_sativus_NC_024469 ......... .......C.. .......... .......... .......... .........

Ricotia_aucheri_NC_039956 ......... .......... ..G....... .A........ .......... .........

Ricotia_carnosula_NC_039957 ......... .......... .......... ...T...... .......... .........

Ricotia_cretica_NC_039958 ......... .......... ......T... ...T...... .......... .........

Ricotia_davisiana_NC_039959 ......... .......... .......... .......... .......... .........

Ricotia_isatoides_NC_039960 .......G. .......... .......... .......... .......... .........

Ricotia_lunaria_NC_039961 ......... T......... .......... .......... .......... .........

Schrenkiella_parvula_NC_028726 ......... ....T..... .......... .......... .......... .........

Sinalliaria_limprichtiana_NC_034287 ......... .......... ..A....... .......... .......... .........

Sinapis_arvensis_NC_035303 ......... .......C.. ......G... .......... .......... .........

Sisymbrium_irio_NC_037838 ......... .......... .......... .......... .......... .........

Solms-laubachia_eurycarpa_NC_034359 ......... .......... .......... .......... .......... .........

Thlaspi_arvense_NC_034362 ......... .......... ..T....... ....T..... ..C....... .........

###### Figure S7 Schematic representation of the genomic organization of *rps12* gene in *A. thaliana*

The LSC, IRb, SSC and IRa regions are shown in the middle of the plot. One copy of coding sequence for exon 1 and two copies of coding sequences for exons 2 and 3 are shown. The exon 1 and the exon2 and 3 that are coded on the positive strand in the IRa region, are joined to form transcript 1. Similarly, the exon 1 and the exons 2 and exon 3 that are coded on the negative strand in the IRb regioin are joined to form transcript 2. Arrows indicate the direction from 5’ to 3’.


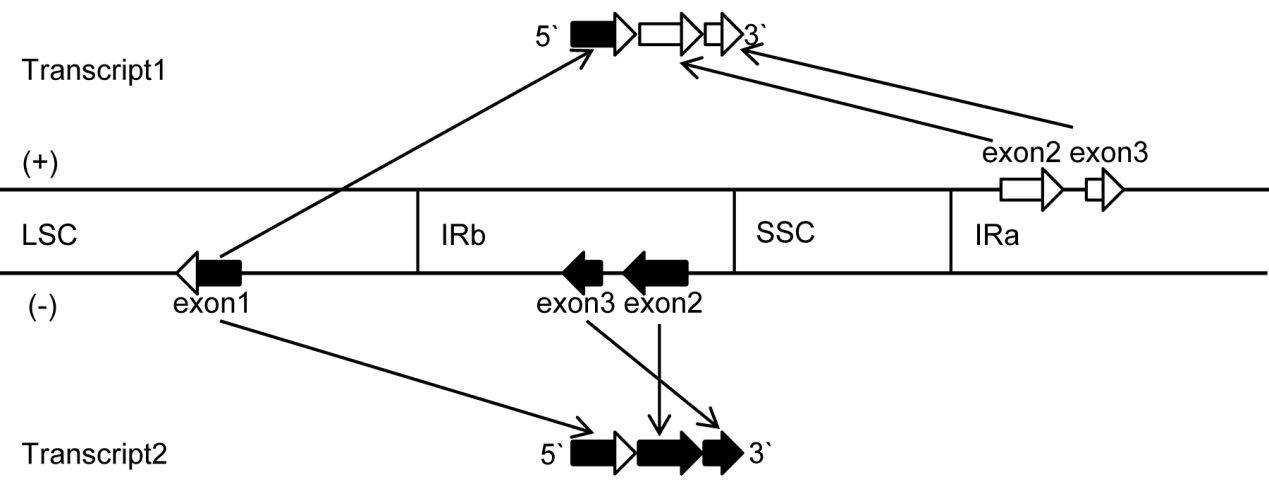


###### Figure S8 Alignment and statistics of the last exon of gene *rps12* from 2544 plastome sequences.

The patterns and the corresponding frequencies are shown. “.”: the nucleotide is the same as that found in the most abundant sequence. “.”: identical; “-”: indel.


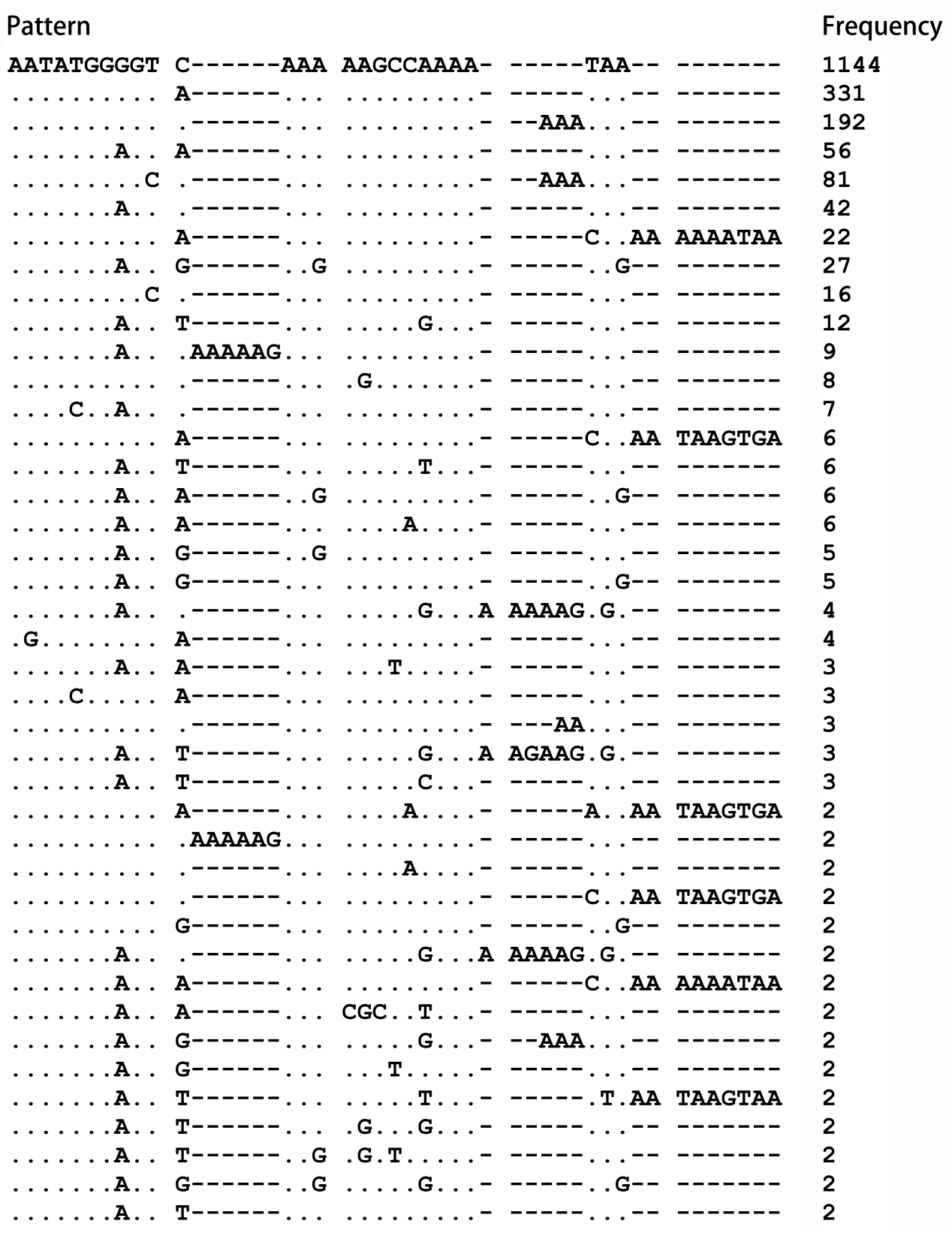


###### Figure S9 Alignment of the last 26 bases of the CDS and 50 bp 3’ UTR downstream of the stop codon of the gene *rps12* from 75 Brassicaceae plants.

The species names and accession numbers from GenBank are shown on the left. The corresponding sequences are shown on the right. The sequence on the top is the reference sequence. The last 26 bases in the CDS are in bold and underlined . “.”: the nucleotide is the same as that found in the reference sequence.

Aethionema_arabicum_NC_034367  **AATATGGGGT CAAAAAGCCA AAATAA**AAGA TTTGAGCCCT TATAAAAAGA AAACAGATTC TTGAACCCCT TTCACG

Aethionema_cordifolium_NC_009265 .......... .......... .......... .......... .......... .......... .......... ......

Aethionema_grandiflorum_NC_009266 .......... .......... .......... .......... .......... .......... .......... ......

Arabidopsis_arenicola_NC_030346 .......... .......... .......... .......... .......... .......... .......... ......

Arabidopsis_arenosa_NC_029334 .......... .......... .......... .......... .......... .......... .......... ......

Arabidopsis_cebennensis_NC_029335 .......... .......... .......... .......... .......... .......... .......... ......

Arabidopsis_croatica_NC_030347 .......... .......... .......... .......... .......... .......... .......... ......

Arabidopsis_halleri_NC_034366 .......... .......... .......... .......... .......... .......... .......... ......

Arabidopsis_lyrata_NC_034365 .......... .......... .......... .......... .......... .......... .......... ......

Arabidopsis_lyrata_subsp._lyrata_NC_034379.......... .......... .......... .......... .......... .......... .......... ......

Arabidopsis_neglecta_NC_030348 .......... .......... .......... .......... .......... .......... .......... ......

Arabidopsis_pedemontana_NC_029336 .......... .......... .......... .......... .......... .......... .......... ......

Arabidopsis_petrogena_NC_030349 .......... .......... .......... .......... .......... .......... .......... ......

Arabidopsis_suecica_NC_030350 .......... .......... .......... .......... .......... .......... .......... ......

Arabidopsis_thaliana_NC_000932 .......... .......... .......... .......... .......... .......... .......... ......

Arabidopsis_umezawana_NC_030351 .......... .......... .......... .......... .......... .......... .......... ......

Arabis_flagellosa_NC_037475 .......... .......... .......... .......... .......... .......... .......... ......

Arabis_hirsuta_NC_009268 .......... .......... .......... .......... .......... .......... .......... ......

Barbarea_verna_NC_009269 .......... .......... .......... .......... .......... .......... .......... ......

Biscutella_baetica_NC_039952 .......... .......... .......... .......... .......... .......... .......... ......

Biscutella_lyrata_NC_039953 .......... .......... .......... .......... .......... .......... .......... ......

Brassica_napus_NC_016734 .......... .......... .......... .......... .......... .......... .......... ......

Brassica_napus_NC_016734_27_3 .......... .......... .......... .......... .......... .......... .......... ......

Brassica_nigra_NC_030450_27_3 .......... .......... .......... .......... .......... .......... .......... ......

Braya_humilis_NC_035515 .......... .......... .......... .......... .......... .......... .......... ......

Bunias_erucago_NC_036110 .......... .......... .......... .......... .......... .......... .......... ......

Bunias_orientalis_NC_036111 .......... .......... .......... .......... .......... .......... .......... ......

Camelina_sativa_NC_029337 .......... .......... .......... .......... .......... .......... .......... ......

Capsella_bursa-pastoris_NC_009270 .......... .......... .......... .......... .......... .......... .......... ......

Capsella_grandiflora_NC_028517 .......... .......... .......... .......... .......... .......... .......... ......

Capsella_rubella_NC_027693 .......... .......... .......... .......... .......... .......... .......... ......

Crucihimalaya_wallichii_NC_009271 .......... .......... .......... .......... .......... .......... .....T.... ......

Draba_nemorosa_NC_009272 .......... .......... .......... .......... .......... .......... .......... ......

Draba_oreades_NC_037760 .......... .......... .......... .......... .......... .......... .......... ......

Eutrema_botschantzevii_NC_029379 .......... .......... .......... .......... .......... .......... .......... ......

Eutrema_halophilum_NC_029378 .......... .......... .......... .......... .......... .......... .......... ......

Eutrema_heterophyllum_NC_028728 .......... .......... .......... .......... .......... .......... .......... ......

Eutrema_salsugineum_NC_028170 .......... .......... .......... .......... .......... .......... .......... ......

Eutrema_yunnanense_NC_028727 .......... .......... .......... .......... .......... .......... .......... ......

Heldreichia_bupleurifolia_NC_039954 .......... .......... .......... .......... .......... .......... .......... ......

Hesperis_matronalis_NC_035511 .......... .......... .......... .......... .......... .......... .......... ......

Hesperis_sylvestris_NC_035512 .......... .......... .......... .......... .......... .......... .......... ......

Isatis_tinctoria_NC_028415 .......... .......... .......... .......... .......... .......... .......... ......

Lepidium_meyenii_NC_034363 .......... .......... .......... .......... .......... .......... .......... ......

Lepidium_virginicum_NC_009273 .......... .......... .......... .......... .......... .......... .......... ......

Lobularia_libyca_NC_035513 .......... .......... .......... .......... .......... .......... .......... ......

Lobularia_maritima_NC_009274 .......... .......... .......... .......... .......... .......... .......... ......

Lunaria_rediviva_NC_039955 .......... .......... .......... .......... .......... .......... .......... ......

Matthiola_incana_NC_034358 .......... .......... .......... .......... .......... .......... .......... ......

Megacarpaea_delavayi_NC_034360 .......... .......... .......... .......... .......... .......... .......... ......

Megadenia_pygmaea_NC_034357 .......... .......... .......... .......... .......... .......... .........C ......

Morettia_canescens_NC_035514 .......... .......... .......... .......... .......... .......... .......... ......

Nasturtium_officinale_NC_009275 .......... .......... .......... .......... .......... .......... .......... ......

Neotorularia_korolkowii_NC_034361 .......... .......... .......... .......... .......... .......... .......... ......

Olimarabidopsis_pumila_NC_009267 .......... .......... .......... .......... .......... .......... .......... ......

Orychophragmus_diffusus_NC_033498 .......... .......... .......... .......... .......... .......... .......... ......

Orychophragmus_hupehensis_NC_033500 .......... .......... .......... .......... .......... .......... .......... ......

Orychophragmus_taibaiensis_NC_033499 .......... .......... .......... .......... .......... .......... .......... ......

Pachycladon_cheesemanii_NC_021102 .......... .......... .......... .......... .......... .......... .......... ......

Pachycladon_enysii_NC_018565 .......... .......... .......... .......... .......... .......... .......... ......

Pugionium_cornutum_NC_030516 .......... .......... .......... .......... .......... .......... .......... ......

Pugionium_dolabratum_NC_030515 .......... .......... .......... .......... .......... .......... .......... ......

Raphanus_sativus_NC_024469 .......... .......... .......... .......... .......... .......... .......... ......

Ricotia_aucheri_NC_039956 .......... ........A. .......... .......... .......... .......... .......... ......

Ricotia_carnosula_NC_039957 .......... .......... .......... .......... .......... .......... .......... ......

Ricotia_cretica_NC_039958 .......... .......... .......... .......... .......... .......... .......... ......

Ricotia_davisiana_NC_039959 .......... .......... .......... .......... .......... .......... .......... ......

Ricotia_isatoides_NC_039960 .......... .......... .......... .......... .......... .......... .......... ......

Ricotia_lunaria_NC_039961 .......... .......... .......... .......... .......... .......... .......... ......

Schrenkiella_parvula_NC_028726 .......... .......... .......... .......... .......... .......... .......... ......

Sinalliaria_limprichtiana_NC_034287 .......... .......... .......... .......... .......... .......... .......... ......

Sinapis_arvensis_NC_035303 .......... .......... .......... .......... .......... .......... .......... ......

Sisymbrium_irio_NC_037838 .......... .......... .......... .......... .......... .......... .......... ......

Solms-laubachia_eurycarpa_NC_034359 .......... .......... .......... A......... .......... .......... .......... ......

Thlaspi_arvense_NC_034362 .......... .......... .......... .......... .......... .......... .......... ......

###### File S1 CDS and protein sequences validated or corrected based on the mapping of RNA-seq data to the plastome sequences of 43 species

###### File S2 Genome-wide RNA-editing site identification using RNA-editing site analysis pipeline from CPGAVAS2

###### File S3 RNA-editing site identification in *ndh*B gene using RNA-editing site analysis pipeline from CPGAVAS2

###### File S4 Annotation results of five tools for the plastome of *A. thaliana*

###### File S5 The sequence of a newly sequenced plastome of *Glechoma longituha*

###### File S6 Annotation results of five tools for a newly sequenced plastome of *G. longituha*
